# A novel long noncoding RNA SP100-AS1 induces radioresistance of colorectal cancer via sponging miR-622 and stabilizing ATG3

**DOI:** 10.1038/s41418-022-01049-1

**Published:** 2022-08-17

**Authors:** You Zhou, Yingjie Shao, Wenwei Hu, Jinping Zhang, Yufang Shi, Xiangyin Kong, Jingting Jiang

**Affiliations:** 1 Tumor Biological Diagnosis and Treatment Center, The Third Affiliated Hospital of Soochow University, Changzhou, 213003 China; 2 Jiangsu Engineering Research Center for Tumor Immunotherapy, Changzhou, 213003 China; 3 Institute of Cell Therapy, Soochow University, Changzhou, 213003 China; 4 Department of Radiation Oncology, The Third Affiliated Hospital of Soochow University, Changzhou, 213003 China; 5 Department of Oncology, The Third Affiliated Hospital of Soochow University, Changzhou, 213003 China; 6 Institutes of Biology and Medical Sciences, Soochow University, Suzhou, 215123 China; 7 The First Affiliated Hospital of Soochow University, State Key Laboratory of Radiation Medicine and Protection, Institutes for Translational Medicine, Soochow University Medical College, Suzhou, 215123 China; 8 CAS Key Laboratory of Tissue Microenvironment and Tumor, Shanghai Institute of Nutrition and Health, Chinese Academy of Sciences, Shanghai, 200031 China

**Keywords:** Oncogenes, Cancer genomics

## Abstract

Although radiotherapy is an essential modality in the treatment of colorectal cancer (CRC), the incidence of radioresistance remains high clinically. Long noncoding RNAs (lncRNAs) reportedly play critical roles in CRC radioresistance by regulating genes or proteins at the transcriptional or post-translational levels. This study aimed to identify novel lncRNAs involved in radioresistance. We found that SP100-AS1 (lncRNA targeting antisense sequence of SP100 gene) was upregulated in radioresistant CRC patient tissues using RNA-seq analysis. Importantly, knockdown of SP100-AS1 significantly reduced radioresistance, cell proliferation, and tumor formation in vitro and in vivo. Mechanistically, mass spectrometry and bioinformatics analyses were used to identify the interacting proteins and microRNAs of SP100-AS1, respectively. Moreover, SP100-AS1 was found to interact with and stabilize ATG3 protein through the ubiquitination-dependent proteasome pathway. In addition, it could serve as a sponge for miR-622, which targeted ATG3 mRNA and affected autophagic activity. Thus, lncRNA SP100-AS1 could act as a radioresistance factor in CRC patients *via* RNA sponging and protein stabilizing mechanisms. In conclusion, the present study indicates that SP100-AS1/miR-622/ATG3 axis contributes to radioresistance and autophagic activity in CRC patients, suggesting it has huge prospects as a therapeutic target for improving CRC response to radiation therapy.

## Introduction

Colorectal cancer (CRC) is one of the most common gastrointestinal malignant tumors and ranks third in the world in terms of malignant tumor incidence [1, 2]. The 5-year survival rate for colorectal cancer ranges from 10 to 90%, depending on the stage of colorectal cancer [3–5]. Overwhelming evidence substantiates that postoperative chemoradiotherapy for rectal cancer reduces local recurrence and improves overall survival [6, 7]. However, in clinical practice, much heterogeneity surrounds interindividual differences in sensitivity to radiotherapy [8, 9]. Therefore, the quest for new biomarkers to predict sensitivity to radiotherapy, develop personalized radiotherapy strategies, and provide individualized treatment for CRC patients is of great clinical significance.

Long noncoding RNAs (lncRNAs) have received much attention in recent years for their role in radiotherapy resistance in malignant tumors [10–13]. LncRNA is a noncoding RNA with a length greater than 200 nucleotides, which is mainly involved in radiotherapy resistance of CRC through epigenetic, transcriptional, and post-transcriptional regulation of gene expression [14, 15]. According to studies, the abnormal sequence and spatial structure [16–19], the abnormal expression level, and the abnormal interaction with binding proteins of lncRNAs are all associated with the occurrence of human diseases such as cancer [20], degenerative neurological diseases [21, 22], and a variety of metabolic diseases [23]. The up-regulation or down-regulation of specific lncRNA in tumor cells can trigger the apoptosis of tumor cells or increase the sensitivity of tumor cells to apoptosis-inducing dependent therapy, such as radiation-induced DNA damage therapy, which provides a new idea for the study of tumor radiotherapy resistance [24–27]. Guo et al. previously identified MALAT1, a novel lncRNA that could regulate radioresistance in colorectal cancer via miR-101-3p sponging [28]. Moreover, they found that MALAT1 was upregulated in radioresistant cancer cell lines, and down-regulation of MALAT1 could inhibit cell proliferation and metastasis through the apoptosis pathway. H19, an intergenic lncRNA, has been shown to be essential in promoting cell differentiation, proliferation, and various cancer therapy resistance [10, 29]. A previous study identified that H19 modified the radio and chemo-sensitivity of cardiac carcinoma cells by interacting with miR-130a-3p and miR-17-4p [30]. Additionally, Ma et al. found that H19 could affect the development and induction of hepatocellular carcinoma by targeting presenilin 1 (PSEN1) [31]. As a result, the critical role of lncRNAs in cancer oncogenesis and radioresistance is well documented.

Autophagy is a process that is temporarily triggered by cellular stress such as DNA damage, in which some damaged proteins or organelles are encapsulated by autophagic vesicles of double-membrane structures and transported to lysosomes for degradation [32–36]. Mechanistically, autophagy depends on a conserved E1-E2-E3 tri-enzyme cascade that catalyzes LC3 protein lipidation, in which the central enzyme is the E2, ATG3. ATG3 receives LC3 from E1 and forms a reactive ATG3-LC3 intermediate. Then LC3 is transferred from the ATG3 catalytic site to phosphatidylethanolamine (PE) lipid molecules in a reaction catalyzed by E3 enzymes [37–39]. Functionally, this process may promote the survival of tumor cells after chemotherapy [40] or radiotherapy [41] and therefore be associated with radiation therapy resistance [42, 43]. More evidence has shown that cancer cells rely on autophagy to avoid radiotherapy-induced DNA damage and reduce cellular apoptosis, so suppressing autophagy in cancer cells might improve cancer cell sensitivity to radiotherapy [44, 45]. Han et al. demonstrated the role and mechanism of lncRNA small nucleolar RNA host gene 14 (SNHG14) in cisplatin-resistant colorectal cancer and confirmed that there was a direct interaction between miR-186 and ATG14. Overexpression of miR-186 mimics and ATG14 gene knockout inhibited the expression of autophagy-associated protein LC3B, while ATG14 overexpression significantly restored their effect. Despite these findings, the radiation-resistant function of lncRNAs in colorectal cancer has received little attention.

Herein, bioinformatics analyses were used to screen out the differences between the radiotherapy sensitive and resistant colorectal cancer tissues. We provided compelling evidence that lncRNA TCONS_0027179 was significantly upregulated in colorectal cancer tissues as well as radiation-resistant tissues. We named this lncRNA SP100-AS1 since it targets the antisense sequence of the SP-100 gene. Notably, we also uncovered the role of SP100-AS1 in modulating CRC cell proliferation and radiosensitivity. Furthermore, our findings shed light on the mechanisms underlying the regulatory role of lncRNAs on CRC radiation sensitivity.

## Materials and methods

### CRC tissue specimen

A total of 44 patients including 22 radiosensitive and 22 radioresistant, who underwent surgery at the Third Affiliated Hospital of Soochow University from 2018 to 2019, were selected for the present study. The collected tissue specimens were stored in liquid nitrogen at −80 °C. All human tissue-related experiments were approved by the Ethical Committee of the Third Affiliated Hospital of Soochow University according to the Declaration of Helsinki. All patients provided informed consent before study enrollment.

### RNA-sequencing analysis

Total RNA from 16 CRC tissues obtained from 8 radiosensitive and 8 radioresistant patients were subjected to HiSeq RNA-Seq. Sequencing reads were mapped to the human genome using Tophat. For lncRNAs, the transcriptome from each dataset was assembled independently using Cufflinks. All transcriptomes were pooled and merged to generate a final transcriptome using Cuffmerge. Differential expression analysis was performed using the DESeq package. *P* values < 0.05 were considered statistically significant. All sequencing processes and analyses were performed by Shanghai OE Biotech Co., Ltd. (Shanghai, China).

### Cell lines

Colorectal cancer cell lines HCT116, SW480, LS174T, CT26, HT29, and LoVo were purchased from American Type Culture Collection (ATCC). Human cell lines were cultured in RPMI-1640 medium supplemented with 10% Fetal Bovine Serum and 1% penicillin-streptomycin. All human cell lines tested negative for mycoplasma. All cells were maintained at 37 °C and 5% CO_2_.

### Xenograft mouse model

All the animal experiments were performed in strict accordance with the principles and procedures approved by the Ethical Committee of Animal Experiments of the Third Affiliated Hospital of Soochow University. A total of 5 × 10^6^ transfected HCT116 cells were subcutaneously injected into six-week-old male nude mice. Mice were randomly assigned to no irradiation (IR) or IR groups (2 Gy). When the tumor size reached about 90 mm^3^, xenograft tumors of the IR groups received local tumor IR with a fractionated dose of 2 Gy every other day for 10 days. Tumor volumes were measured every five days and calculated with the formula: (length × width^2^) / 2.

### IR

Treatment of IR was given at indicated dose with a linear accelerator (Varian 2300EX, Varian, Palo Alto, CA) that generated 6MV X-ray. Briefly, monolayer cells were exposed to X-ray at different time courses, and cells were harvested immediately after IR. The control group cells were also placed into an X-ray generator under the same conditions without IR.

### Transfection

The SP100-AS1 siRNA sequences were transfected into SW480 and HCT116 cells using lipofectamine 2000 reagent (Thermo Fisher) according to the instruction manual. Subsequently, the cells were collected after 48 h of transfection for use in subsequent experiments.

### Apoptosis assay

SW480 cells were seeded in 6-well plates for 24 h to measure apoptosis and then transfected with the indicated siRNA or plasmids. After 24–36 h, the cells were collected, washed with ice-cold PBS 3 times, and gently resuspended in a 500 μL binding buffer. Thereafter, the cells were stained with Annexin V/FITC. Subsequently, propidium iodide (PI) was added to the buffer and incubated for another 10 min in the dark. Finally, the stained cells were analyzed using CytoFLEX (BECKMAN COULTER).

### Real-time qPCR

Total RNA was extracted from the cells using Trizol according to the operating manual. Then, cDNA was generated using the Superscript First-Strand cDNA Synthesis Kit (18080-051, Invitrogen) and RT-qPCR was performed using Power SYBR Green PCR master mix (Thermo Fisher) on LightCycle 480 Real-Time PCR system (Roche).

### Cell proliferation assay

The cell proliferation activity was evaluated using an MTT assay. After being transfected with the indicated siRNA, cells were seeded onto 96-well plates and incubated for another 48 h at 37 °C in 5% CO_2_. Subsequently, the medium was changed and the cells of each well were incubated with MTT medium (1 mg/mL) for 2 h. Spectrometry reading at 540 nM was carried out, and further analysis was conducted. All experiments were performed independently in triplicate at least.

### Western blot

Cells were washed with ice-cold PBS and lysed in RIPA lysis buffer containing protease/phosphatase inhibitor cocktail (CST) and PMSF, then centrifuged to remove insoluble sections. The concentration of proteins was quantified using the Pierce BCA Protein Assay Kit (Thermo Fisher). Equal proteins were resolved by SDS-PAGE gel, transferred onto polyvinylidene fluoride (PVDF) membranes (Bio-Rad), and then probed with γ-H2AX (Abcam, ab81299), LC3 (Sigma, L8918), P62 (Abcam, ab109012), ATG3 (Abcam, ab108251), HA (Cell Signaling Technology, #5017), and Flag antibodies (Cell Signaling Technology, #14793) at 4 °C overnight. Blots were imaged using a Licor Odyssey imaging system, and the band intensities were normalized to GAPDH.

### Luciferase reporter assay

SP100-AS1 and ATG3 3’URT sequence wild type (WT) and mutation (Mut) were synthesized and constructed in a pGL3 vector. The luciferase reporter plasmids were transfected into SW480 and HCT116 cells, and the normalized Renilla vector (pRL-TK) and the indicated siRNA were co-expressed simultaneously. The luciferase activity was measured with the dual-luciferase reporter assay kit (Promega) following the manufacturer’s protocol.

### RNA immunoprecipitation (RIP) assay

SW480 and HCT116 cells were lysed in RIP buffer. After centrifugation, the cell lysates were incubated with anti-ATG3 and control IgG antibodies at 4 °C overnight. The magnetic beads were added into lysates and incubated for another 1 h at room temperature. The magnetic beads were washed 3 times using RIP buffer and precipitated RNAs were eluted and subjected to RT-qPCR.

### Pull-down assay

The sense and antisense chain of SP100-AS1 were synthesized and labeled with biotin. The pull-down assay was performed using streptavidin magnetic beads (Beyotime). The beads were washed with RIP buffer 3 times, and binding proteins were detected using silver staining followed by SDS-PAGE.

### Statistical analysis

All data were presented as the mean ± standard error of the mean (SEM) from at least three independent experiments. Differences between groups were evaluated with Student’s *t*-test and one-way ANOVA analysis. Graphs were processed with GraphPad Prism software. *P* values < 0.05 were considered statistically significant. Sample sizes of all experiments were predetermined according to our experience. No sample was excluded from the analyses. Animals were not randomly assigned, but the sex, strain, and age of the mice were the same, and the data analysis was single-blinded. Investigators were not blinded to the group allocation during the experiment and outcome assessment.

## Results

### SP100-AS1 upregulation is associated with CRC radioresistance

**Fig. 1:**
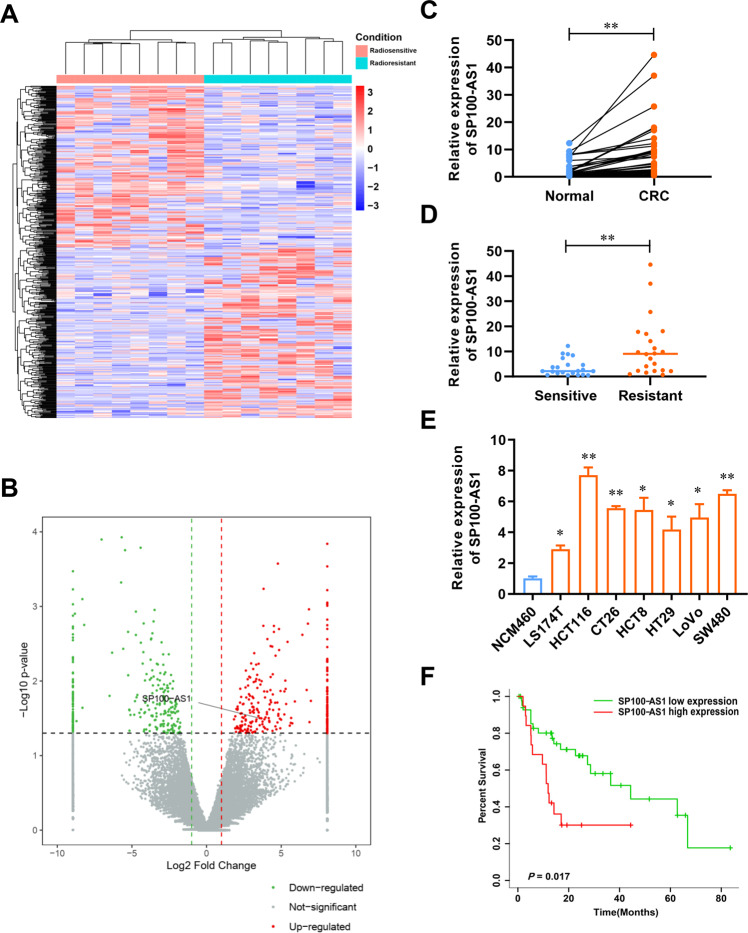
SP100-AS1 was upregulated in CRC and positively related to radiotherapy. **A** The expression profile of lncRNA in radiosensitive and radioresistant colorectal cancer tissues. **B** SP100-AS1 was upregulated in colorectal cancer tissues, as shown by RNA-seq. **C** The relative expression of SP100-AS1 in colorectal cancer tissues (CRC, *n* = 44) and normal tissues (Normal, *n*  =  44). **D** The relative expression of SP100-AS1 in radiotherapy-sensitive (Sensitive, *n* = 22) and resistant patients’ colorectal tissues (Resistant, *n* = 22). **E** The relative expression of SP100-AS1 in normal human colon mucosal epithelial cell line NCM460 and a series of human colorectal cancer cell lines (*n* = 3). **F** The relationship between SP100-AS1 levels and overall survival of colorectal cancer patients, obtained from the GEPIA database. *P < 0.05, **P < 0.01 compared with the indicated group.

The differential expression of lncRNAs in CRC tissues was analyzed and compared between 8 radiosensitive and 8 radioresistant patients using RNA-seq analysis (Fig. 1A). The results demonstrated that 267 lncRNAs were upregulated in radioresistant samples compared to radiosensitive samples (Fig. 1B, Supplementary Table 1), of which SP100-AS1 was significantly overexpressed. Next, RT-qPCR analysis showed that SP100-AS1 expression in CRC tissues (*n* = 44) was significantly higher than in adjacent normal tissues (Fig. 1C). Taken together, the above findings suggested that SP100-AS1 might play an important role in CRC progression. Moreover, we hypothesized that SP100-AS1 could regulate the radiosensitivity of CRC based on its abnormal expression in radioresistant tissues. Accordingly, we collected CRC tissues from patients with radiosensitive and radioresistant clinical characteristics. As shown in Fig. 1D, the radioresistant CRC tissues exhibited higher expression of SP100-AS1. Taken together, our findings suggested that SP100-AS1 was involved in CRC progression and radioresistance. Using RT-qPCR, the endogenous SP100-AS1 expression was investigated in normal human colon mucosal epithelial cell NCM460 and CRC cell lines, including LS177T, HCT116, CT26, HCT8, HT29, LoVo, and SW480 (Fig. 1E). The results showed that CRC cells harbored higher expression of SP100-AS1 in comparison to normal epithelial cell lines, especially in HCT116 and SW480 cell lines, which were used in subsequent experiments. Interestingly, high expression of SP100-AS1 was associated with poor survival of CRC patients (Fig. 1F). Collectively, these findings indicated that lncRNA SP100-AS1 overexpression correlated with radioresistance and poor survival in CRC.

### SP100-AS1 knockdown enhances CRC radiosensitivity in vitro

**Fig. 2:**
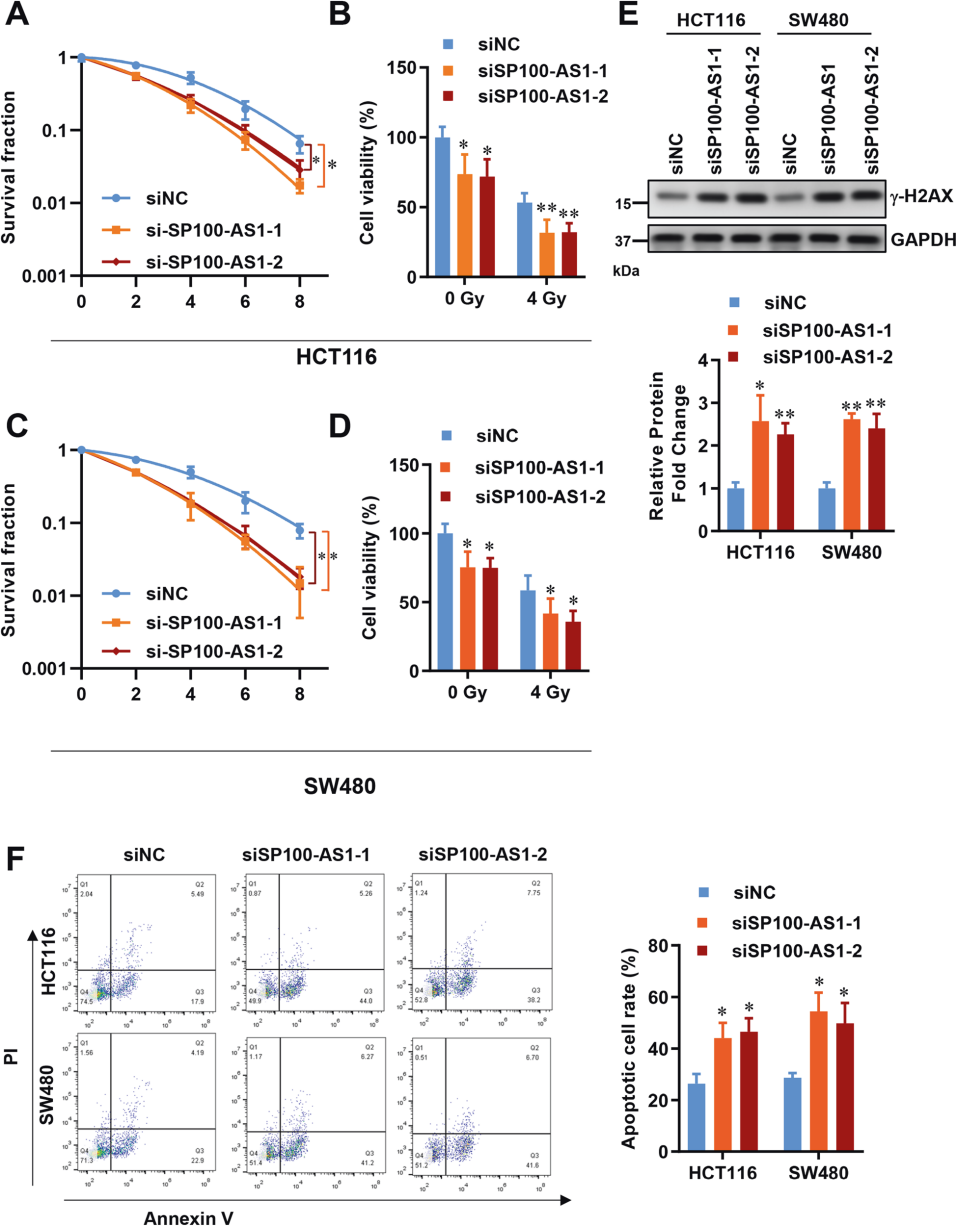
Downregulation of SP100-AS1 exacerbated radio-induced cell death. **A** The cellular survival curves of HCT116 following SP100-AS1 knockdown using siRNA. **B** The cell viability of HCT116 cells at a radiation dose of 4 Gy. **C** The cellular survival curves of SW480 following SP100-AS1 knockdown. **D** The cell viability of SW480 following 4 Gy of IR. **E** The expression of γ-H2AX in HCT116 and SW480 cell lines following SP100-AS1 knockdown and the gray intensity histogram of γ-H2AX expression. **F** Cell apoptosis was evaluated by flow cytometry, and the apoptotic cell percentage was statistically analyzed. *n* = 3, *P < 0.05, **P < 0.01 compared with the indicated group.

Having established the abnormal expression pattern of SP100-AS1 in CRC patient tissues and cell lines, we investigated how this lncRNA modulates tumor cell proliferation and survival. As previously mentioned, SP100-AS1 was most abundantly expressed in HCT116 and SW480 cells in a dose-dependent manner (Figs. 1E,  S1). A clonogenic assay was conducted to determine the effect of SP100-AS1 on radiosensitivity in HCT116 and SW480 cells. Notably, when SP100-AS1 was downregulated, the radiosensitivity of these two cell lines was significantly increased (Fig. 2A, C). Moreover, the viability of HCT116 and SW480 cells at dose under 4 Gy was investigated, showing that SP100-AS1 silencing reduced cell proliferation and viability (Fig. 2B, D).

It is widely acknowledged that radiation therapy causes double-strand DNA breaks, which must be repaired before further tumor growth. This finding emphasizes the need to develop a new approach to block DNA repair and stop tumor growth or at least slow them down to prolong patient survival. Blocking DNA damage repair is a particularly attractive strategy for treating CRCs since they are highly resistant to radiation. As expected, SP100-AS1 inhibition could induce the expression of γ-H2AX, a significant marker of DNA damage (Fig. 2E). Next, we analyzed the impact of SP100-AS1 on cell apoptosis. Annexin V/PI double staining demonstrated that SP100-AS1 deletion significantly increased apoptotic response in HCT116 cells compared to the control (Fig. 2F). This suggests that SP100-AS1 is involved in CRC radiation-induced DNA damage and apoptosis. Therefore, SP100-AS1 knockdown could increase the radiosensitivity of CRC.

### SP100-AS1 confers significant radioresistance in vivo

**Fig. 3:**
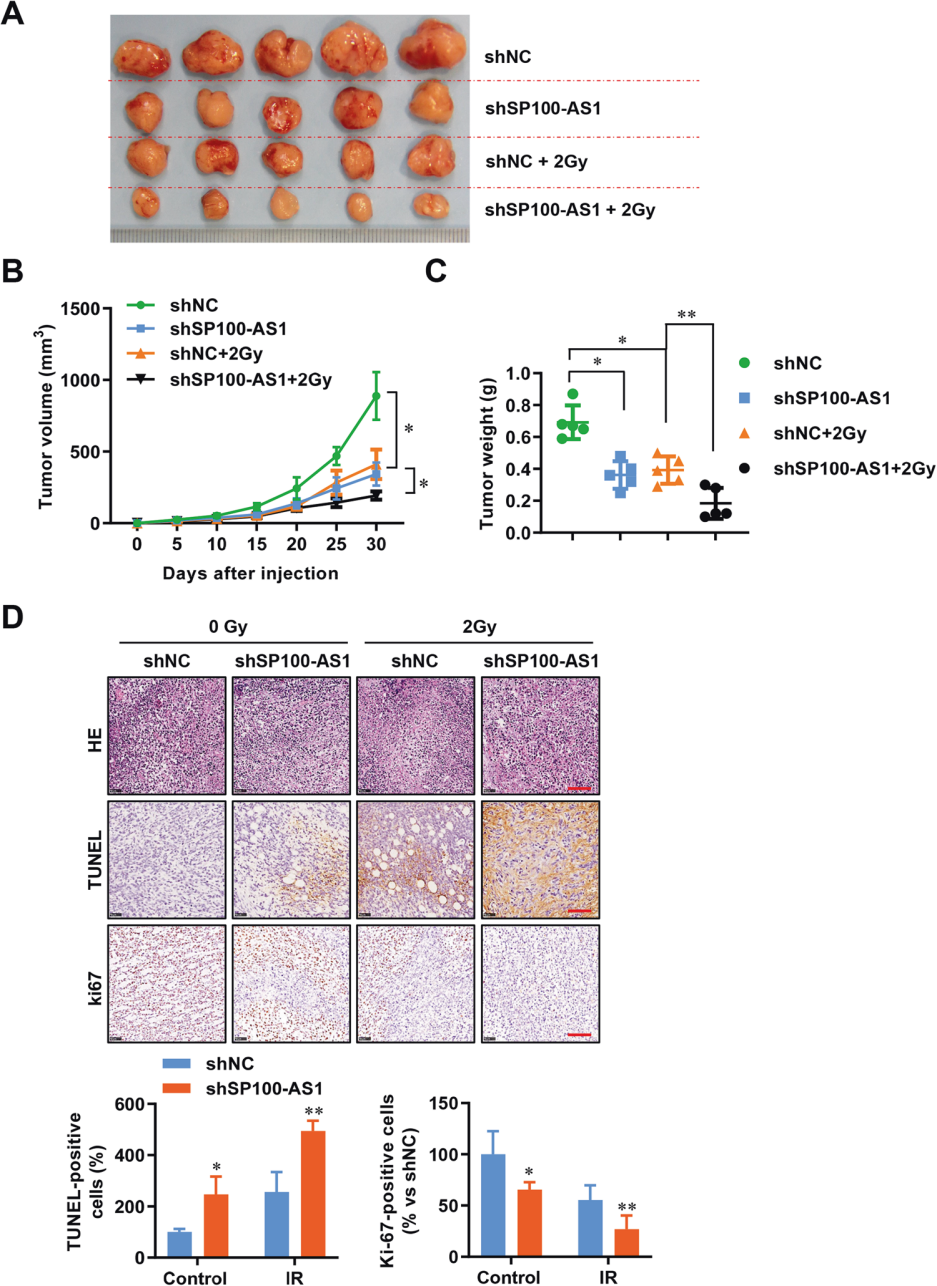
SP100-AS1 downregulation reduced the growth of irradiated-CRC cells in vivo. **A** SP100-AS1 was stably knocked down in HCT116, and cells were injected subcutaneously into the axilla of nude mice. 2 Gy IR every other day for 10 days was given until the tumors reached 90 mm^3^. The image of excised tumors is presented. **B** The growth curves of tumors from (**A**). **C** The tumor weight from (**A**). **D** The images following TUNEL staining and representation of relative Ki67 expression. Scale bars = 100 μm. *n* = 5, *P < 0.05, **P < 0.01 compared with the indicated group.

Next, we investigated the effect of SP100-AS1 in vivo by establishing a xenograft tumor model using BALB/c nude mice. As shown in Fig. 3A, SP100-AS1 was stably knocked down by lentiviral infection of HCT116 cells, and the inoculated xenografts showed reduced tumor growth compared to the control group. Moreover, after IR, HCT116 tumor cells with SP100-AS1-knockdown grew slower compared to the control group (Fig. 3A). After fractionated IR, silencing of SP100-AS1 could further slow down the growth of CRC tumors (Fig. 3B). At the end of the experiment, the weight of the tumors was measured (Fig. 3C). Subsequently, transferase-mediated dUTP nick-end labeling (TUNEL) assay showed that knocking down SP100-AS1 caused more DNA damage under 2 Gy radiation and reduced Ki67 staining, suggesting that SP100-AS1 downregulation inhibited CRC growth speed, especially when exposed to radiation (Fig. 3D). Taken together, these results demonstrated that SP100-AS1 silencing could enhance the radiosensitivity of CRC in vitro and in vivo.

### SP100-AS1 increases colorectal cancer cell radioresistance activity through the autophagy pathway

**Fig. 4:**
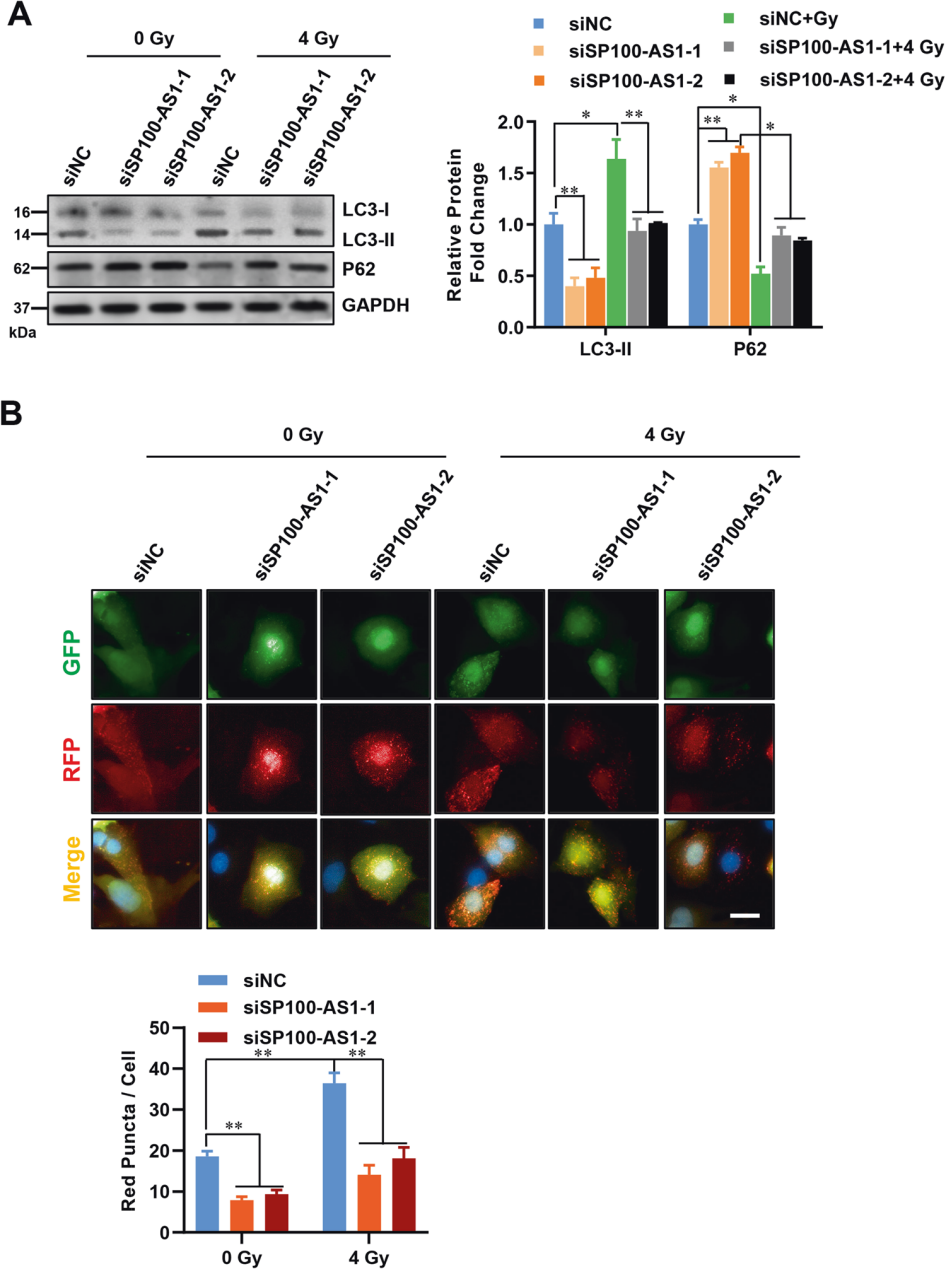
SP100-AS1 regulated cell proliferation through the autophagy pathway in HCT116 cells. **A** HCT116 cells were irradiated at 4 Gy, and relative protein fold change of autophagy-related proteins LC3 and p62 were quantified. **B** Fluorescence images of HCT116 cells treated in (**A**) infected with LC3-GFP-RFP overexpressing lentivirus and subjected to 4 Gy radiation. Quantification of average dots per cell of RFP and GFP signals in each cellular condition was presented. Scale bars = 20 μm. *n* = 3. *P < 0.05, **P < 0.01 compared with the indicated group.

Activation of the autophagy pathway plays an important role in radiation resistance in various tumors, including colorectal cancer [46]. To explore whether the autophagy pathway was involved in the radiation resistance in HCT116 and SW480 cells, we quantified the protein expression of LC3-II and p62/SQSTM1, which are well-recognized autophagic flux markers of autophagosome formation [47, 48], after SP100-AS1 knockdown. The results indicated that LC3-II was decreased, and p62 protein levels were increased (Figs. 4A,  S2A). Moreover, we used the LC3-GFP-RFP report system to assess autophagosome formation [49]. LC3 is a membrane-bound protein fused with green and red fluorescent proteins. Unlike RFP, GFP is unstable in an acidic environment. Accordingly, the RFP puncta or RFP/GFP ratio was calculated to measure the autophagic flux. Following SP100-AS1 knockdown, HCT116 cells were infected with lentivirus overexpressing LC3-GFP-RFP and treated with the indicated level of radiation. Consistent with previous findings, the induction of autophagy was much weaker, and the autophagic flux was significantly reduced under radiation treatment compared with the control group (Figs. 4B,  S2B). Moreover, western blot and IHC analyses showed that p62 protein expression was significantly reduced, and LC3-II protein level was strikingly increased in CRC xenograft tumors with IR treatment, which was reversed by silencing of SP100-AS1 (Fig. S3A, B). Therefore, these results concluded that radiation could induce autophagy in HCT116 cells, and silencing SP100-AS1 could alleviate this effect.

### SP100-AS1 regulates the autophagy pathway through ATG5 and Beclin1

**Fig. 5:**
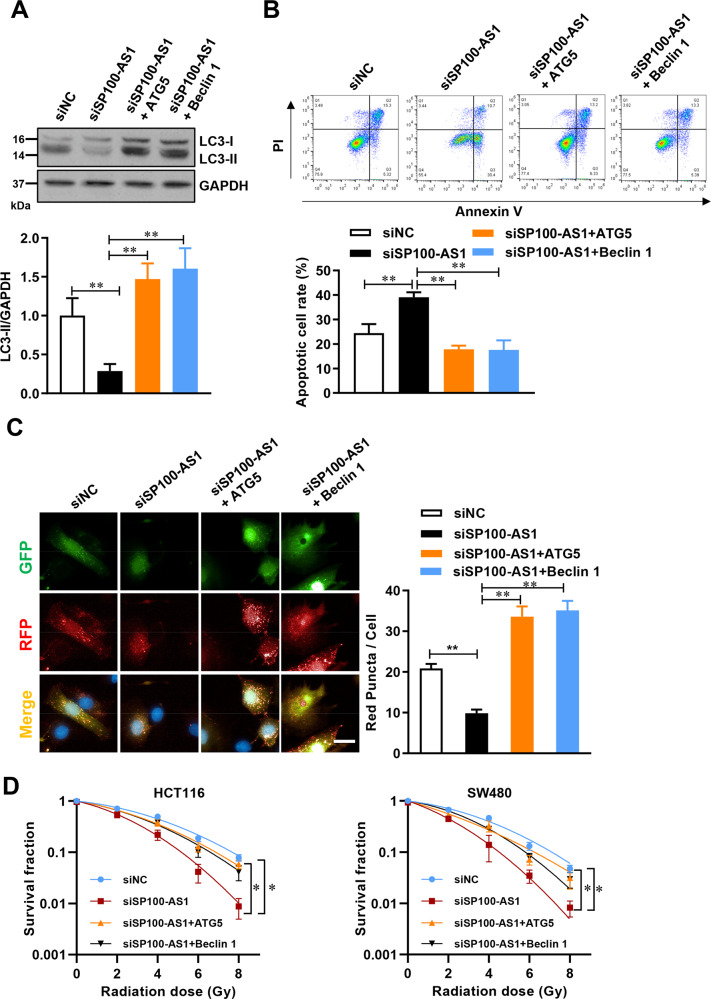
SP100-AS1 regulation of autophagy was rescued by ATG5 and Beclin1 overexpression. **A** HCT116 cells overexpressed ATG5 and Beclin1 following SP100-AS1 knockdown. The relative expression of LC3 was detected. **B** Histogram representing the apoptosis rates of HCT116 in **A** measured by flow cytometry. **C** Fluorescence images of HCT116 cells in **A** infected with LC3-GFP-RFP lentivirus. The red puncta level per cell was statistically analyzed. **D** Both HCT116 (left panel) and SW480 (right panel) cells overexpressed ATG5 and Beclin1 following SP100-AS1 knockdown. The cellular survival curve of the indicated cells was presented. Scale bars = 20 μm. *n* = 3, *P < 0.05, **P < 0.01 compared with the indicated group.

ATG5 and Beclin1 are vital upstream regulators in the macro-autophagy pathway, and overexpression of ATG5 and Beclin1 has been reported to directly activate the autophagy signaling pathway. Therefore, we investigated whether SP100-AS1-regulated autophagy could be rescued by overexpressing ATG5 and Beclin1. Following knockdown of SP100-AS1 in HCT116 and SW480 cells, ATG5 and Beclin1 were upregulated, and protein expression of LC3-II was measured. As shown in Fig. 5A and Fig. S4A, autophagy flux reduced by SP100-AS1 downregulation was rescued by the upregulation of ATG5 or Beclin1. In addition, the flow cytometry assay was used to quantify apoptosis following Annexin V/PI double staining, which indicated that upregulation of ATG5 and Beclin1 could reduce cell apoptosis induced by knocking down SP100-AS1 (Figs. 5B,  S4B). As previously mentioned, an LC3-GFP-RFP lentivirus reporter was used to identify the autophagic flux density, and the red puncta number per cell was measured. Upregulation of ATG5 or Beclin1 could significantly increase the puncta number following SP100-AS1 knockdown in HCT116 cells (Fig. 5C). Furthermore, we investigated the function and role of ATG5 and Beclin1 on cell proliferation and survival in both HCT116 and SW480 cells. These results showed that either ATG5 or Beclin1 upregulation could rescue the decline in cell proliferation resulting from SP100-AS1 downregulation in these two cell lines (Fig. 5D), implying that SP100-AS1 regulated autophagy through the canonical macro-autophagy pathway.

### SP100-AS1 interacts with ATG3 and regulates its protein stability

**Fig. 6:**
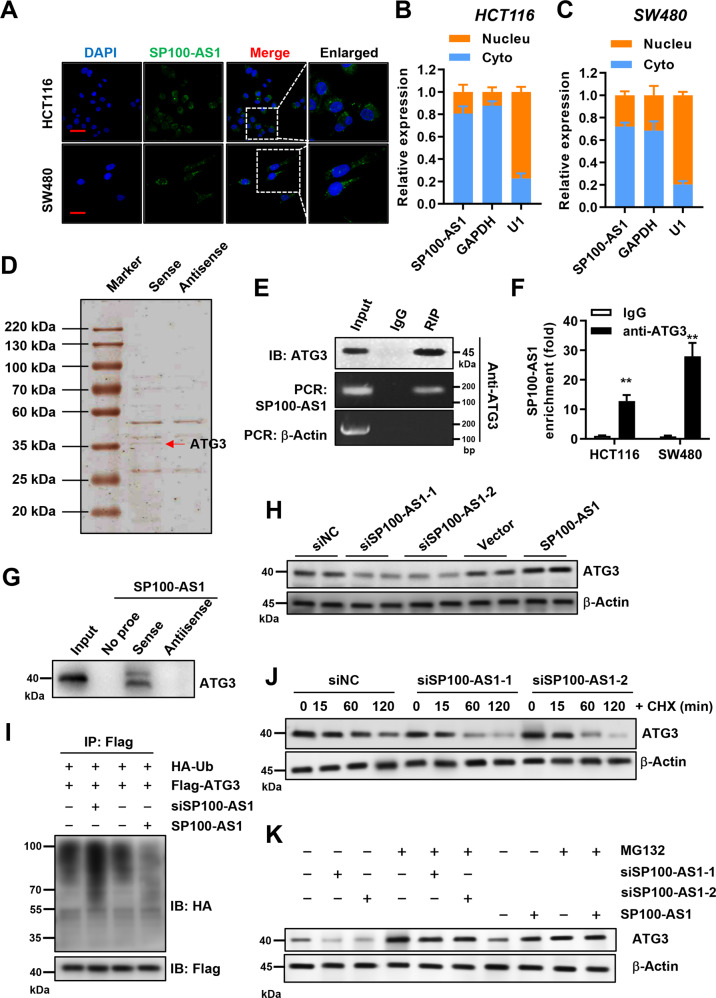
SP100-AS1 interacted with ATG3 and regulated its protein stability. **A** FISH image of SP100-AS1 in HCT116 and SW480 cells. **B** The localization rate of SP100-AS1 in the cytoplasm (Cyto) and the nucleus (Nucleu) of HCT116. **C** The localization rate of SP100-AS1 in the cytoplasm (Cyto) and nucleus (Nucleu) of SW480. **D** Proteins retrieved from the SP100-AS1 pull-down assay were analyzed by mass spectrometric analysis. **E** RIP assay using anti-ATG3 antibodies showed that ATG3 interacted with SP100-AS1 in HCT116 cells. **F** The enrichment level of anti-ATG3 antibodies pulling down SP100-AS1. **G** Western blot analysis of the proteins retrieved from the SP100-AS1 pull-down assay, with its antisense sequence used as a negative control. **H** The ATG3 expression in HCT116 cells following knockdown or overexpression of SP100-AS1. **I** The ubiquitination level of ATG3 was analyzed. **J** HCT116 knockdown SP100-AS1 cells were treated with CHX for the indicated time, and the stability and expression of ATG3 were analyzed. **K** HCT116 cells used in (**H**) were treated with MG132, and the expression of ATG3 was analyzed. Scale bars = 50 μm. *n* = 3, **P < 0.01 compared with the indicated group.

It is well-established that lncRNAs can interact with specific proteins to perform their function [15]. To evaluate whether SP100-AS1 could act by this mechanism, we investigated the subcellular localization of SP100-AS1 in both HCT116 and SW480 cells by the fluorescence in situ hybridization (FISH) assay. Surprisingly, SP100-AS1 exhibited strong fluorescence in the cytoplasm, which indicated that this lncRNA could bind to cytoplasmic proteins and carry out related functions, including cell proliferation and radiation resistance (Fig. 6A, B, C). Next, we performed an RNA pull-down assay to uncover proteins that interact with SP100-AS1. As shown in Fig. 6D, several bands were detected at the molecular weight position of 35 kDa by silver staining (Fig. 6D). Mass spectrometry showed that ATG3 peptide levels were significantly high. Moreover, RIP assay of SP100-AS1 was carried out, and the western blot result showed that SP100-AS1 could specifically interact with ATG3 (Fig. 6E). On the other hand, anti-ATG3 antibodies were used to identify the related binding RNA, and it was shown that SP100-AS1 was significantly enriched in ATG3-bound extract compared with the control IgG group (Fig. 6F). Strictly, western blot analysis of proteins retrieved from the SP100-AS1 pull-down assay confirmed that ATG3 protein specifically bound to the sense sequence of SP100-AS1 but not to the antisense control (Fig. 6G). Taken together, we found that ATG3 was the specific binding protein of SP100-AS1 in vivo and in vitro.

Having established that SP100-AS1 could interact with ATG3, we next sought to investigate whether SP100-AS1 could regulate ATG3 protein stability or function. First, we quantified the protein level of ATG3 after knocking down SP100-AS1 in HCT116 cells. Downregulation and overexpression of SP100-AS1 could significantly reduce and increase ATG3 protein levels, respectively (Fig. 6H). Protein stability is regulated by several pathways and mechanisms, especially the ubiquitination-dependent proteasome degradation signaling pathway [50, 51]. Consistently, it has been shown that ATG3 could be ubiquitinated [52]. Therefore, we assessed whether SP100-AS1 could affect ATG3 protein stability through the proteasome pathway. In HCT116 cells, HA-tagged ubiquitin proteins were overexpressed after transfection with Flag-tagged ATG3 while endogenous SP100-AS1 lncRNA was knocked down. The ubiquitination level of ATG3 was detected using anti-HA antibodies after immunoprecipitating with anti-Flag antibodies. We found that silencing of SP100-AS1 increased the ubiquitination level of ATG3, suggesting that SP100-AS1-mediated ATG3 degradation was involved in ubiquitination-dependent degradation (Fig. 6I). Moreover, we investigated the time course of ATG3 degradation with or without treatment of cycloheximide (CHX), a well-established inhibitor of protein synthesis. Importantly, we found that knockdown of SP100-AS1 could accelerate ATG3 degradation (Fig. 6J). Furthermore, SP100-AS1 was knocked down or overexpressed in HCT116 cells, then treated with the proteasome inhibitor MG132. As shown in Fig. 6K, the degradation of ATG3 protein induced by knocking down SP100-AS1 was rescued. What’s more, overexpression of SP100-AS1 and treatment with MG132 dramatically increased the expression of ATG3. These results confirmed that SP100-AS1 interacted with ATG3 and regulated its protein stability.

### SP100-AS1 serves as a sponge for miR-622 in CRC

**Fig. 7:**
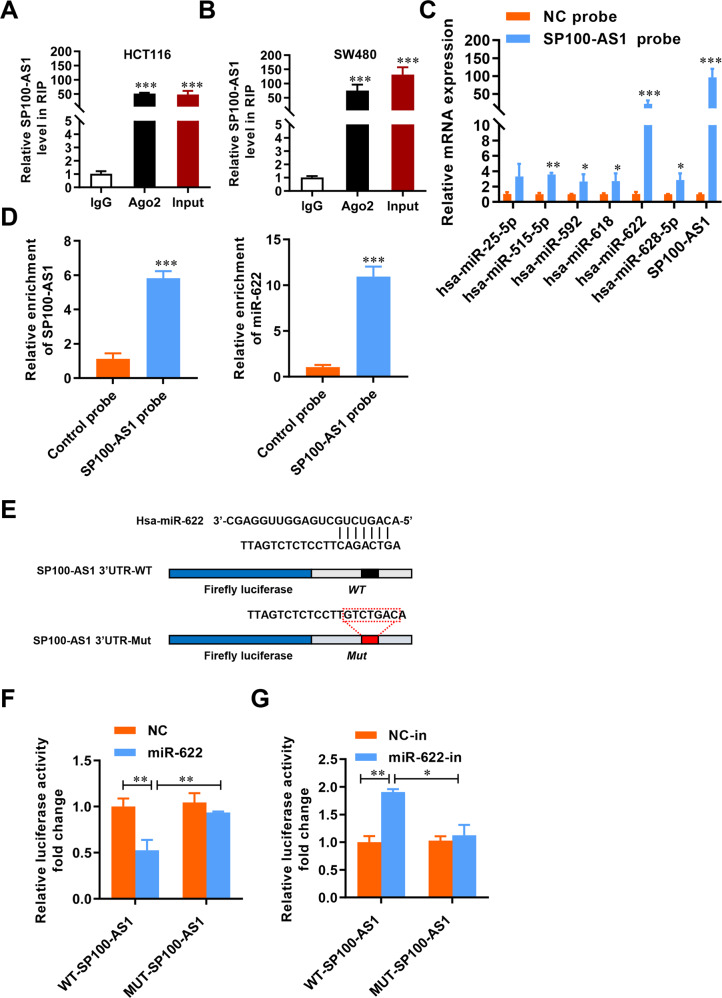
SP100-AS1 serves as a sponge for miR-622 in CRC. **A** RIP assay showed SP100-AS1 interacted with AGO2 in HCT116 cells. **B** SP100-AS1 interacted with AGO2 in SW480 cells. **C** RNA pull-down assay was carried out using an SP100-AS1 probe. **D** The enrichment level of SP100-AS1 and miR-622 using the indicated probes. **E** Bioinformatic analysis screened for miR-622 interaction with wild-type or mutated SP100-AS1. **F** RNA pull-down assay for the luciferase activity of SP100-AS1-WT and SP100-AS1-MUT in HCT116 co-transfected with miR-622 mimics. **G** RNA pull-down assay as in (**F**) in HCT116 cells co-transfected with anti-miR-622. *n* = 3, *P < 0.05, **P < 0.01, ***P < 0.001 compared with the indicated group.

To further investigate the mechanism by which SP100-AS1 regulated ATG3 protein expression, we assessed whether SP100-AS1 could serve as a miRNA sponge for ATG3 mRNA. A RIP assay was performed to determine if AGO2, which plays various roles in the miRNA pathway and participates in the miRNA assembly process by producing pre-miRNA, is associated with SP100-AS1 [53, 54]. Interestingly, a significant interaction between AGO2 and SP100-AS1 was detected in both HCT116 and SW480 cell lines (Fig. 7A, B). Next, we sought to determine which miRNA could directly bind to SP100-AS1. We used a lncRNA probe to screen the interaction between several miRNAs and SP100-AS1. SP100-AS1-associated miRNAs were purified by the pull-down assay with specific probes targeting SP100-AS1. The results showed that SP100-AS1 and miR-622 were the most significantly enriched (Fig. 7C). These findings substantiated the interaction between miR-622 and SP100-AS1 in HCT116 cells. As shown in Fig. 7D, SP100-AS1 could significantly bind to miR-622. Taken together, these findings demonstrated that SP100-AS1 served as a miR-622 sponge in CRC.

Furthermore, bioinformatics analysis revealed that the SP100-AS1 3’UTR region contained a miR-622 targeting motif. Thus a luciferase assay system was constructed to detect the regulatory effect of miR-622 on SP100-AS1 mRNA (Fig. 7D). Firefly luciferase cassette was linked to wild-type or mutated SP100-AS1 3’UTR regions. We found that mutated SP100-AS1 3’UTR could not interact with miR-622 (Fig. 7E). The luciferase plasmids were then transfected into HCT116 cells. When miR-622 was overexpressed, the intensity of the luciferase carrying the wild-type region was lower than the mutated type (Fig. 7F). Conversely, inhibition of miR-622 (miR-622-in) induced a remarkable increase in luciferase activity, which was also abolished by binding sites mutation (Fig. 7G). Overall, we corroborated that miR-622 could directly bind to SP100-AS1 and affect its mRNA stability in CRC cells.

### miR-622 targets ATG3 mRNA and regulates autophagic flux

**Fig. 8:**
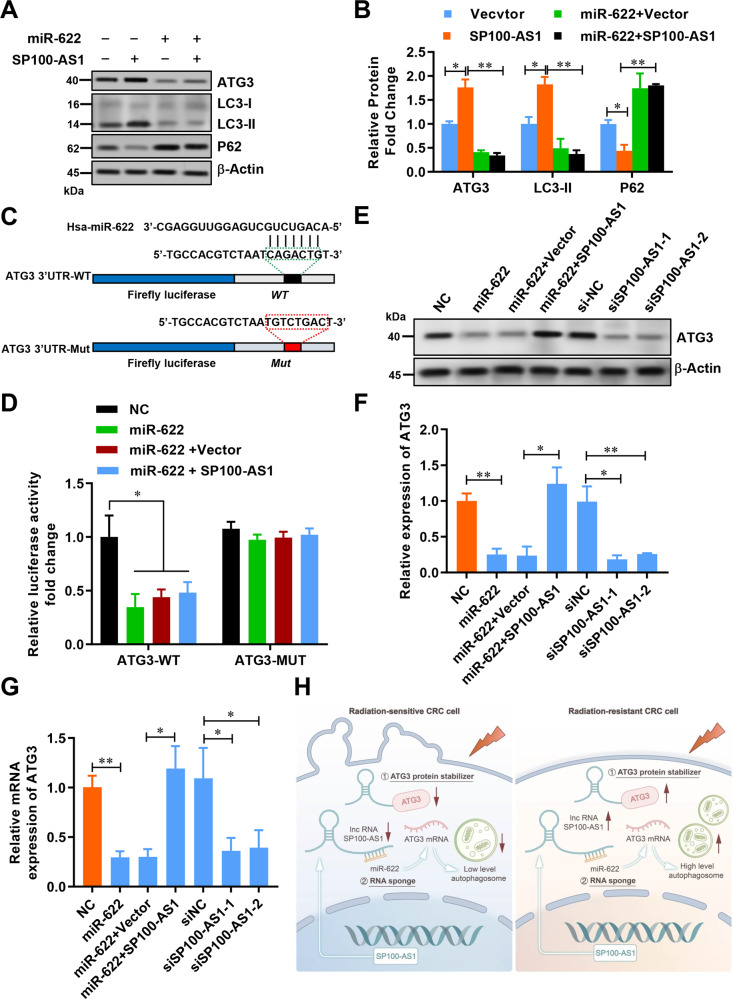
SP100-AS1 regulated ATG3 expression by interacting with miR-622. **A** The indicated protein expression in HCT116 cells overexpressing miR-622 and SP100-AS1. **B** The relative protein change in (**A**). **C** Bioinformatic analysis screened for miR-622 interaction with wild-type and mutant ATG3 3’UTR. **D** The luciferase assay of ATG3 3’UTR in HCT116 cells co-transfected with miR-622 and SP100-AS1. **E** The expression of ATG3 in HCT116 cells co-transfected with miR-622 and SP100-AS1. **F** The relative expression of ATG3 was represented using a histogram. **G** The relative mRNA level of ATG3. **H** Schematic illustration depicting a proposed model of the molecular mechanism of SP100-AS1 in initiating radioresistance in human colorectal cancer. *n* = 3, *P < 0.05, **P < 0.01 compared with the indicated group.

Considering that SP100-AS1 sponged miR-622 and downregulated autophagic flux, we sought to investigate whether miR-622 could affect the autophagy pathway. We overexpressed miR-622 or SP100-AS1 into HCT116 cells. Notably, overexpression of SP100-AS1 decreased p62 and upregulated LC3-II expression, while upregulating miR-622 increased p62 and inhibited LC3-II expression (Fig. 8A, B). Interestingly, ectopic expression of miR-622 in SP100-AS1 overexpressed HCT116 cells could restore p62 and LC3-11 expression (Fig. 8A, B). Therefore, we hypothesized that SP100-AS1 could function as a sponge for miR-622 and prevent its effect on ATG3 mRNA, which could stabilize ATG3 and promote autophagic flux. The ATG3 3’UTR region was analyzed using in silico methods to validate this hypothesis. Surprisingly, one targeting motif of miR-622 was detected. Next, a luciferase assay was conducted in which firefly luciferase cassettes were inserted in the front of the wild-type and mutated ATG3 3’UTR regions (Fig. 8C). As shown in Fig. 8D, overexpression of miR-622 could decrease the expression of wild-type luciferase, while co-transfection with SP100-AS1 could rescue the luciferase activity. In contrast, co-transfection of a plasmid carrying the mutated ATG3 3’UTR region could not restore luciferase activity (Fig. 8D). The endogenous expression of ATG3 in HCT116 cells was then investigated, and the results showed that miR-622 significantly decreased ATG3 expression at the mRNA and protein levels, while co-expression with SP100-AS1 could restore ATG3 expression. As expected, SP100-AS1 knockdown using siRNA could also decrease ATG3 mRNA levels (Fig. 8E, F, G).

To illustrate the biological role of SP100-AS1/miR-622 axis in CRC cells following IR, the cell viability, colony survival ability and apoptotic rate were further investigated. The results showed that co-transfection of SP100-AS1 with miR-622 or siATG3 significantly reversed SP100-AS1-mediated increase in CRC cell viability and colony survival ability (Fig. S5A, B). Moreover, the decreased cell viability and colony survival ability induced by miR-622 mimics could be restored by SP100-AS1 or ATG3 overexpression (Fig. S5C, D). Furthermore, the alteration of the apoptosis rate induced by IR after SP100-AS1 and/or miR-622 overexpression was investigated. Flow cytometry results revealed that co-expression of miR-622 or ATG3 siRNA significantly restored the apoptotic rate compared to cells overexpressing SP100-AS1 (Fig. S5E). MiR-622 mimics could significantly induce apoptosis, and this increase could be reversed by co-expression with SP100-AS1 or ATG3 (Fig. S5F). To further validate the role of ATG3 in miR-622-mediated IR response, HCT116/mock, HCT116/miR-622, or HCT116/miR-622/ATG3 cells were subcutaneously injected into nude mice. Notably, the tumor size derived from miR-622/ATG3-expressing HCT116 cells was larger than those from miR-622-expressing HCT116 cells (Fig. S6A, B, C). Collectively, we demonstrated that lncRNA SP100-AS1 was a key regulating factor in CRC radioresistance, functioning as a sponge for miR-622 and directly promoting the stabilization of ATG3.

## Discussion

Different tissues and organs, particularly tumor tissues, respond differently following radiation exposure [55–57]. Colorectal cancer is a common malignant tumor disease that is clinically treated using a combination of surgery, radiation and chemotherapy [58–60]. With the advent of personalized medicine, several molecular targeting drugs have recently been developed [61]. It is well established that a variety of molecular characteristics of CRC should be considered before selecting the ideal clinical treatment. Ras-family G-proteins, for example, are frequently mutated in CRC, with KRAS mutation occurring in approximately 40% of cases and NRAS mutation occurring in 5–8% of cases [62]. In addition to drug therapy, radiation therapy is frequently used to treat CRC. Since radiotherapy efficacy is dependent on radiosensitivity and radiation therapy tolerance, it is critical to identify specific changes in tumor cells after radiotherapy to locate molecular targets that could increase the anti-tumor effect of radiotherapy in CRC treatment.

Herein, we identified a novel transcript, lncRNA SP100-AS1 that was found to be significantly upregulated in radiation-resistant CRC tissues using bioinformatics analyses. Besides, SP100-AS1 silencing could increase the sensitivity of CRC cells to radiation and further confer an enhanced autophagic flux phenotype. Furthermore, our findings indicated that SP100-AS1 was a mediator of radiation resistance, highlighting it as a promising molecular target in CRC clinical therapy.

Next, we tested the hypothesis that the interaction between SP100-AS1 and ATG3 might affect cell proliferation, radiation-resistant activity, and autophagy flux. First, we looked into how SP100-AS1 affected radiation-induced DNA damage and apoptosis. As previously stated, silencing SP100-AS1 caused DNA damage and cell apoptosis. Second, we speculated on the role of the autophagy pathway in SP100-AS1-regulated radiation resistance. SP100-AS1 was found to reduce CRC radiosensitivity activity both in vitro and in vivo. Thirdly, we mechanistically revealed that SP100-AS1 regulated the autophagy pathway through ATG5 and Beclin1, which were core regulators of the autophagy signaling pathway.

Autophagy is an important catabolic process that is necessary for regulating cell proliferation and tumorigenesis, in which ATG3, as an E2 enzyme, serves as a central regulatory protein in the initiation of LC3 lipidation and autophagosome particles. Moreover, the abnormal expression of ATG3 was shown to be related to autophagic flux and apoptosis. We also found that SP100-AS1 could target ATG3 protein and stabilize its expression by inhibiting the ubiquitination-dependent degradation pathway, as demonstrated by the following: (1) SP100-AS1 interacted with ATG3, (2) downregulation of SP100-AS1 reduced ATG3 expression and accelerated the turnover of ATG3, while SP100-AS1 overexpression had the opposite effect, (3) SP100-AS1 diminished the polyubiquitination of ATG3. As a result, we surmise that SP100-AS1 can regulate the polyubiquitination and degradation of ATG3, but further in-depth studies are required to validate our findings.

Our research also found that SP100-AS1 functioned as a radioresistance mediating factor through an RNA ‘sponging’ mechanism. LncRNAs have been reported to serve as competing endogenous RNAs by functioning as sponges that interact and seclude miRNAs [63]. Based on our findings, we hypothesized that SP100-AS1 might also bind to some miRNAs, sponging their activities and thus targeting complementary mRNAs. Interestingly, we found that SP100-AS1 could sponge miR-622, whose target is ATG3 mRNA, and the crosstalk between SP100-AS1 and miR-622 was found to participate in radiation resistance and proliferation mechanisms of CRC. Next, we further analyzed the mechanism through which miR-622 regulated CRC radiation resistance. Based on bioinformatics analyses, we found that miR-622 targeted and delayed ATG3 turnover. Similarly, western blot results showed that overexpression of miR-622 could decrease ATG3 protein level and increase the expression of autophagy flux marker, LC3-II. Furthermore, SP100-AS1 was able to rescue ATG3 down-regulation caused by miR-622, elucidating the molecular signaling relationship between SP100-AS1, miR-622, and ATG3.

In conclusion, the present study sheds light on two mechanisms, as shown in Fig. 8H. Notably, SP100-AS1 functioned as a protein ‘stabilizer’ by interacting and preventing ATG3 degradation. Meanwhile, SP100-AS1 also acted as an RNA ‘sponge’ for miR-622 to safeguard ATG3 expression. As lncRNAs are a large and diverse class of transcripts that affect gene regulation through a variety of mechanisms, increasing evidence has shown that lncRNAs could bind to proteins and miRNAs synchronously during tumor progression [64, 65]. Hence, we speculated that these two mechanisms were synergistically involved in the biological process of SP100-AS1 in regulating radiotherapy resistance. Certainly, our findings indicate that SP100-AS1/miR-622/ATG3 axis contributes to radioresistance and autophagic flux in CRC, suggesting that it could be a potential target for improving CRC response to radiation therapy.

## Supplementary information

Supplementary legends

Supplementary Figure 1

Supplementary Figure 2

Supplementary Figure 3

Supplementary Figure 4

Supplementary Figure 5

Supplementary Figure 6

Supplementary Table 1

Supplemental Material Original Blots

CDD-21-2807RR-Author-Contribution-Form

CDD-21-2807RR-Author-Contribution-Form-Signed

CDD-21-2807RR-Checklist-Form

## Data Availability

RNA-seq data supporting the results of this study have been deposited in the NCBI GEO database (https://www.ncbi.nlm.nih.gov/geo/query/acc.cgi?acc=GSE186940) under accession number GSE186940.
