# Supplementary material for: A novel long noncoding RNA SP100-AS1 induces radioresistance of colorectal cancer via sponging miR-622 and stabilizing ATG3
Source: Cell Death Differ. 2022 Aug 17;30(1):111–24. doi: 10.1038/s41418-022-01049-1 (PMC9883267; doi:10.1038/s41418-022-01049-1)
Supplement: Supplementary file 1 — Supplementary legends [file 41418_2022_1049_MOESM1_ESM.doc]

**SUPPLEMENTARY LEGENDS**

**Fig. S1 The expression level of SP100-AS1 in HCT116 (A) and SW480 (B) cells.** n=3, **P* < 0.05 compared with the indicated group.

**Fig. S2 SP100-AS1 regulated colorectal cancer cell proliferation through autophagy pathway in SW480 cells.** **A** SW480 cells were irradiated at 4 Gy, and the expressions of autophagy-related proteins LC3 and p62 were analyzed; the relative protein fold change is presented on the right panel. **B** Fluorescence images of SW480 cells treated in (A) were infected using LC3-GFP-RFP overexpressed lentivirus, followed by 4 Gy IR. Quantification of average dots per cell of RFP and GFP signals in each cellular condition was presented. n=3, **P* < 0.05, ***P* < 0.01 compared with the indicated group.

**Fig. S3 Western blot analysis (A) and Representative immunohistochemical images (B) of LC3 and p62 in excised xenograft tumor tissues.** Scale bars: 50 μm. n=3, **P* < 0.05, ***P* < 0.01 compared with the indicated group.

**Fig. S4 SP100-AS1 regulated autophagy was rescued by ATG5 and Beclin1 overexpression.** **A** SW480 cells were overexpressed ATG5 and Beclin1 following SP100-AS1 knockdown. The expression of LC3 was detected and the relative level was presented. **B** The apoptosis rates of SW480 in (A) were measured by flow cytometry, and a histogram representing the apoptosis rate is shown. n=3, **P* < 0.05, ***P* < 0.01 compared with the indicated group.

**Fig. S5 miR-622 reversed the effects of SP100-AS1 on radiosensitivity in CRC cells. A-F** Cells were co-transfected with the indicated vectors/siRNAs/miR-622, after which the cellular survival curves (A and C), the cell viability (B and D) and cell apoptosis (E-F) was measured. n=3, **P* < 0.05, ***P* < 0.01 compared with the indicated group.

**Fig. S6 ATG3 reversed miR-622-indued growth reduction in irradiated-CRC cells *in vivo*. A** The growth of subcutaneous tumor derived from HCT116/Mock, HCT116/miR-622, and HCT116/miR-622/ATG3 cells in BALB/c nude mice. The image of excised tumors was presented. **B** The growth curves of tumors from (A) were measured and illustrated. **C** The tumor weight from (A) was presented. n=5, **P* < 0.05, ***P* < 0.01 compared with the indicated group.

**Supplementary Table 1.** RNA-seq analysis identified a total of 518 differentially expressed lncRNAs in radioresistant versus radiosensitive patients, among which 267 lncRNAs were upregulated, and 251 lncRNAs were downregulated.
