# Supplementary figures and images for: A novel long noncoding RNA SP100-AS1 induces radioresistance of colorectal cancer via sponging miR-622 and stabilizing ATG3
Source: Cell Death Differ. 2022 Aug 17;30(1):111–24. doi: 10.1038/s41418-022-01049-1 (PMC9883267; doi:10.1038/s41418-022-01049-1)

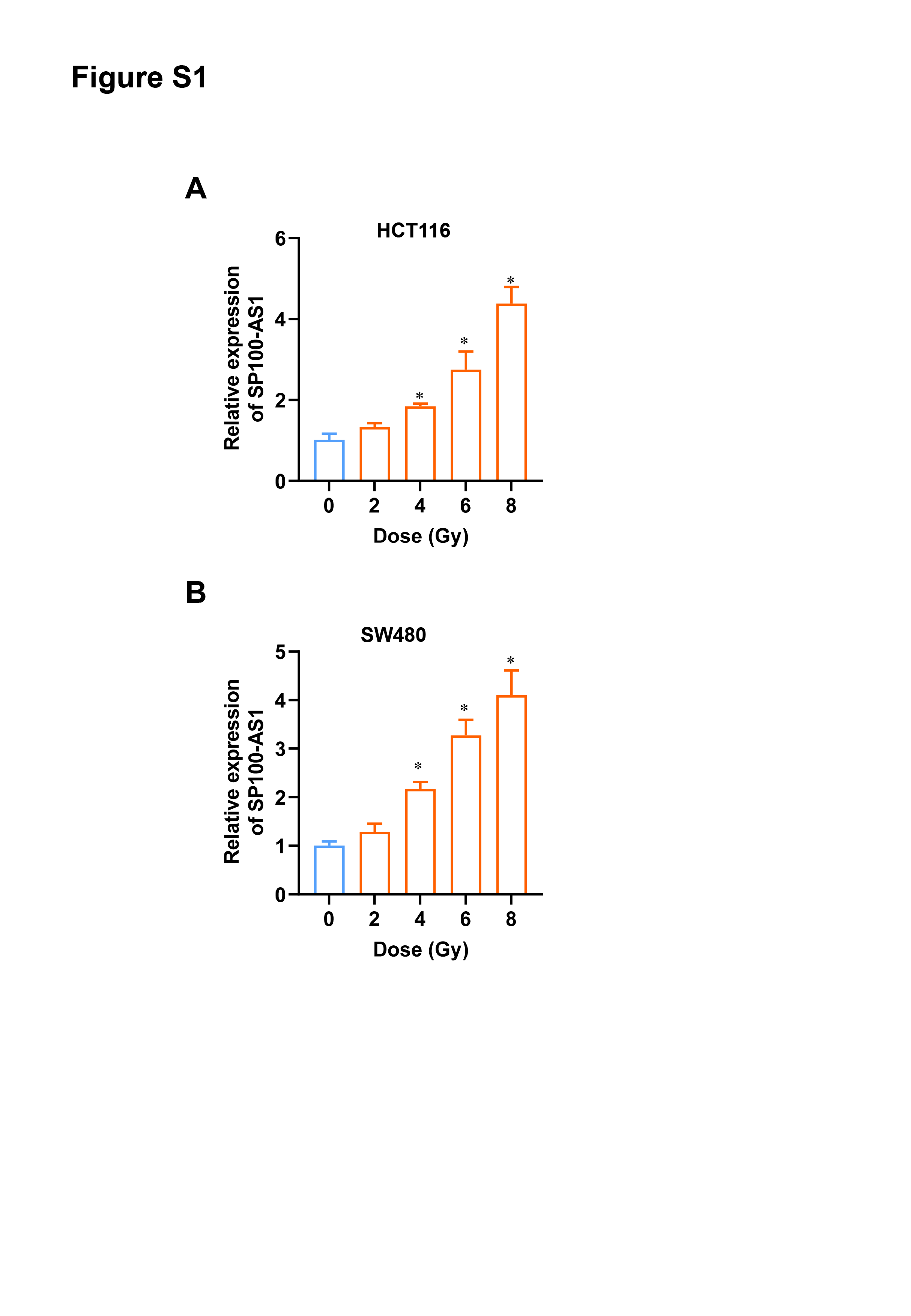

Supplement: Supplementary file 2 — Supplementary Figure 1 [file 41418_2022_1049_MOESM2_ESM.tif]

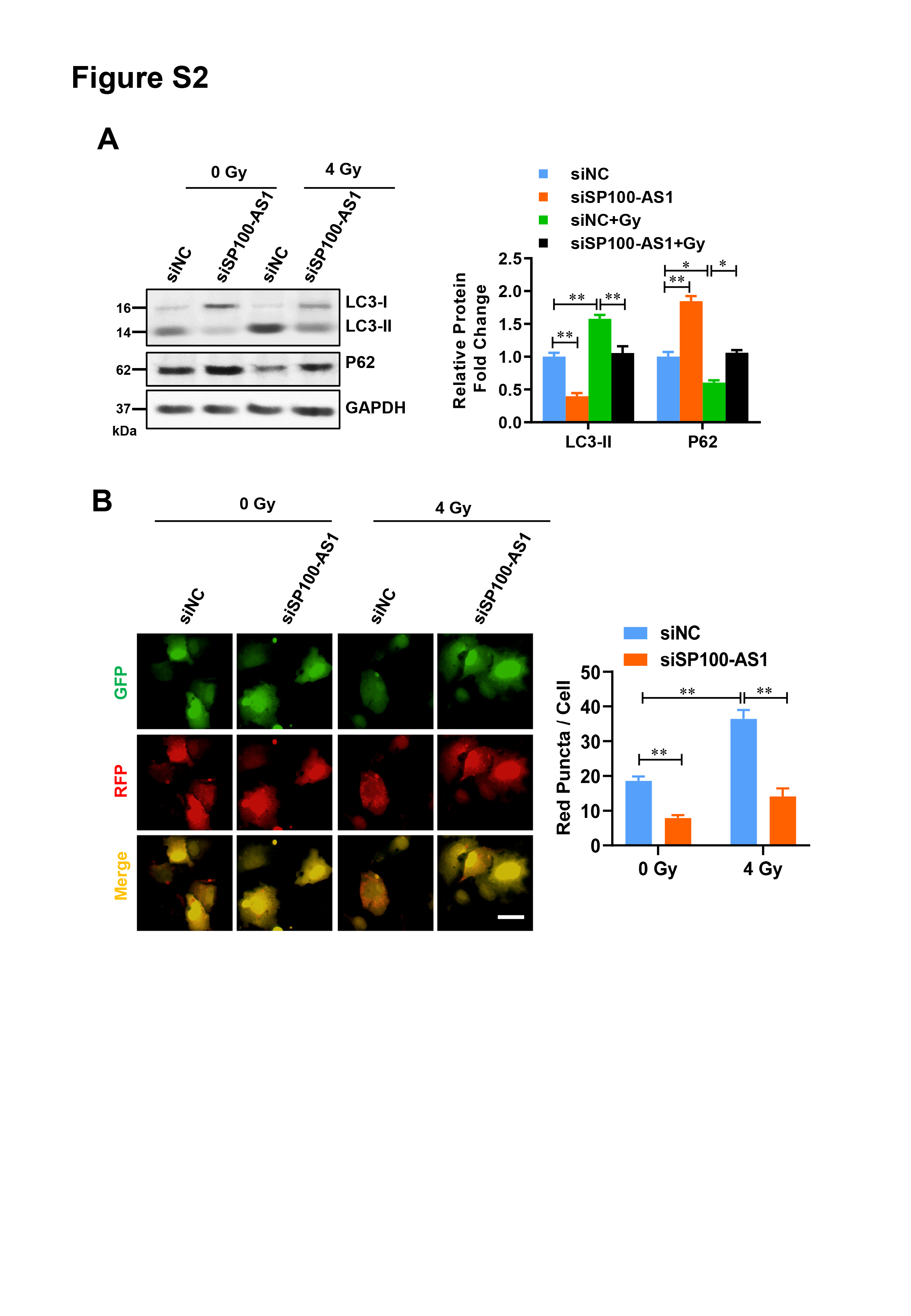

Supplement: Supplementary file 3 — Supplementary Figure 2 [file 41418_2022_1049_MOESM3_ESM.tif]

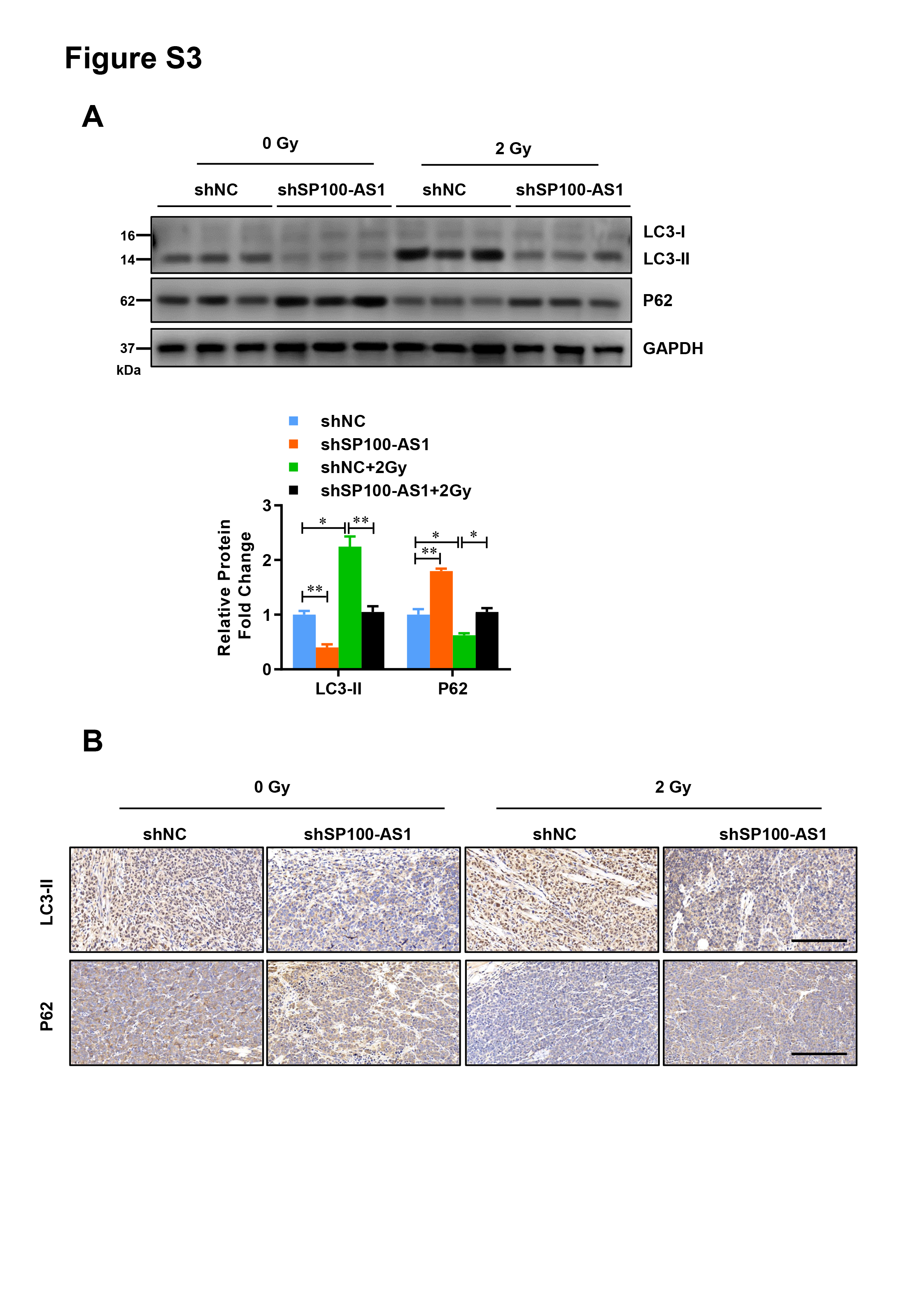

Supplement: Supplementary file 4 — Supplementary Figure 3 [file 41418_2022_1049_MOESM4_ESM.tif]

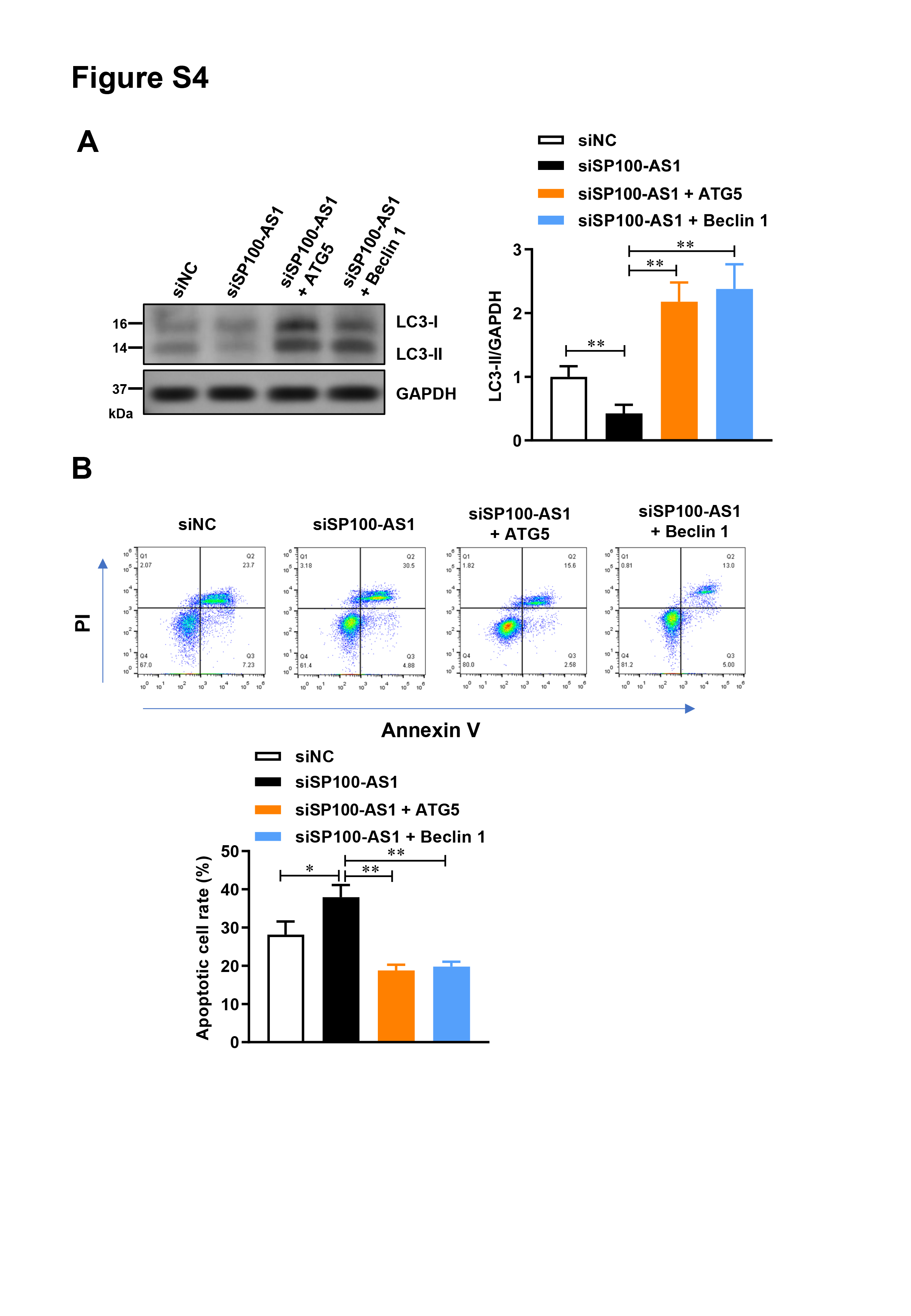

Supplement: Supplementary file 5 — Supplementary Figure 4 [file 41418_2022_1049_MOESM5_ESM.tif]

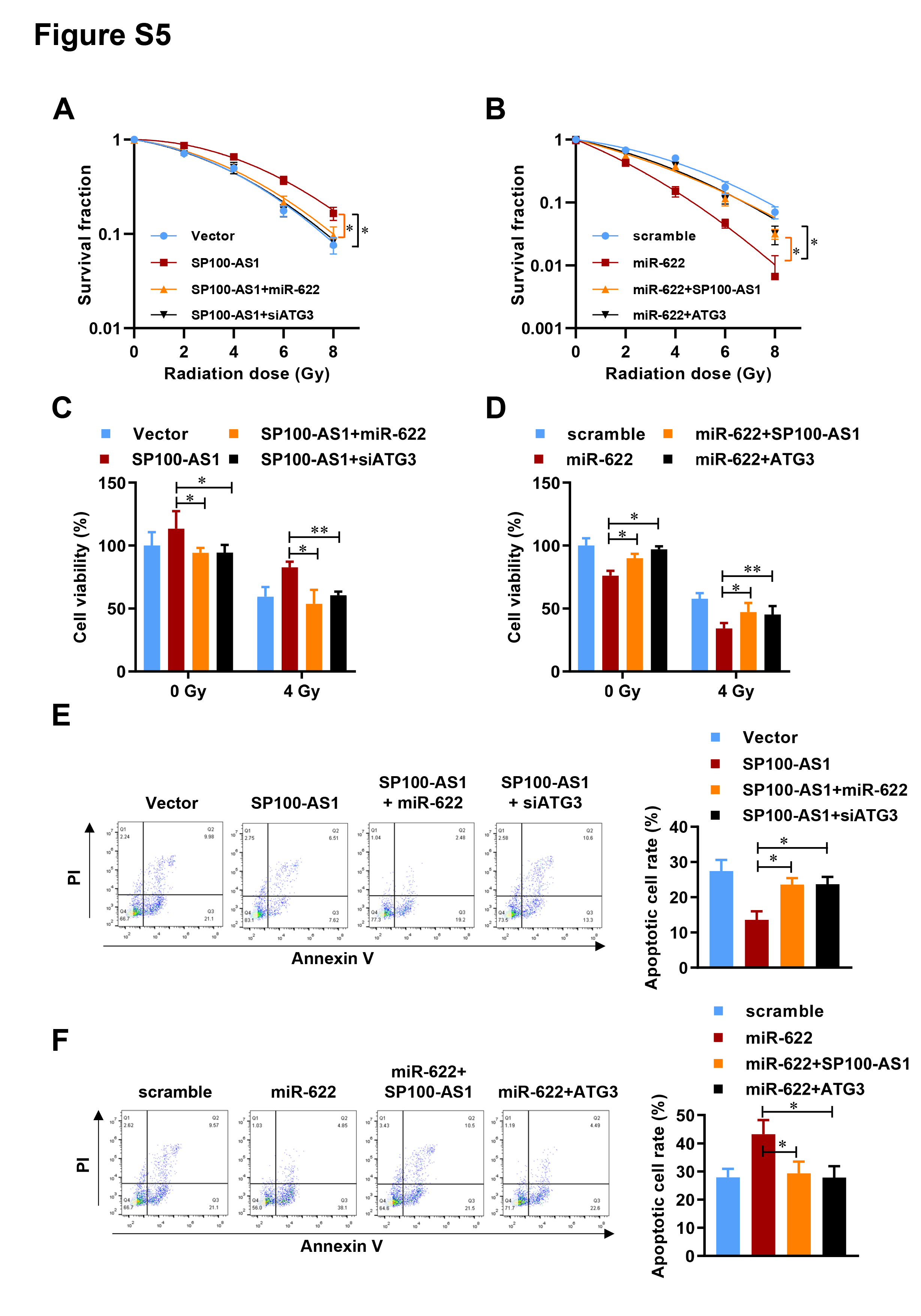

Supplement: Supplementary file 6 — Supplementary Figure 5 [file 41418_2022_1049_MOESM6_ESM.tif]

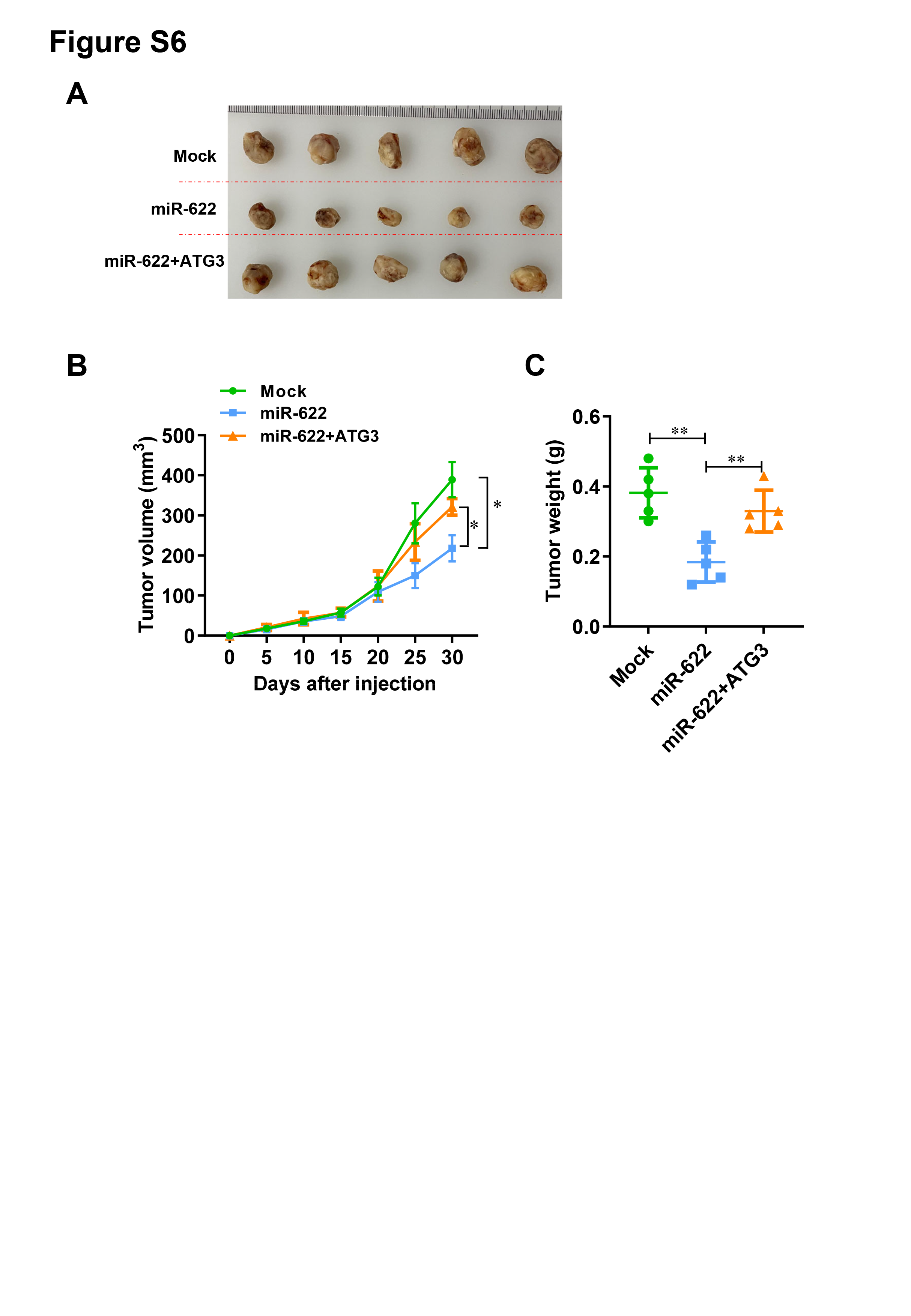

Supplement: Supplementary file 7 — Supplementary Figure 6 [file 41418_2022_1049_MOESM7_ESM.tif]
