# Supplementary material for: A novel long noncoding RNA SP100-AS1 induces radioresistance of colorectal cancer via sponging miR-622 and stabilizing ATG3
Source: Cell Death Differ. 2022 Aug 17;30(1):111–24. doi: 10.1038/s41418-022-01049-1 (PMC9883267; doi:10.1038/s41418-022-01049-1)
Supplement: Supplementary file 9 — Supplemental Material Original Blots [file 41418_2022_1049_MOESM9_ESM.pdf]

Supplemental Material

# – Original Blots

Figure 2E

E

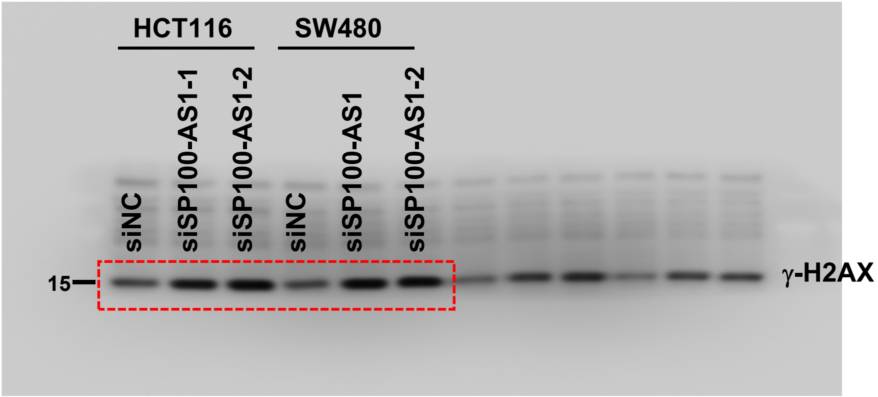
HCT116
SW480
siNC
siSP100-AS1-1
siSP100-AS1-2
siNC
siSP100-AS1
siSP100-AS1-2
15
γ-H2AX

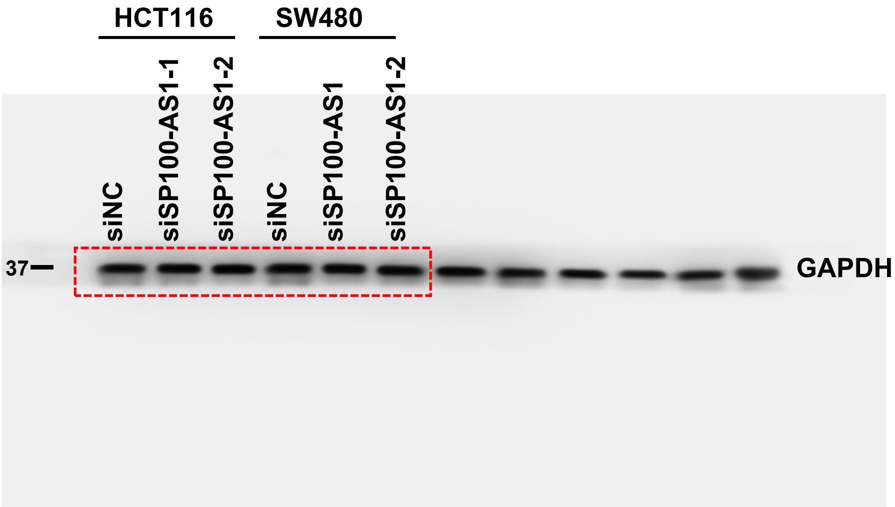
HCT116
SW480
siNC
siSP100-AS1-1
siSP100-AS1-2
siNC
siSP100-AS1
siSP100-AS1-2
37
GAPDH

Figure 4A

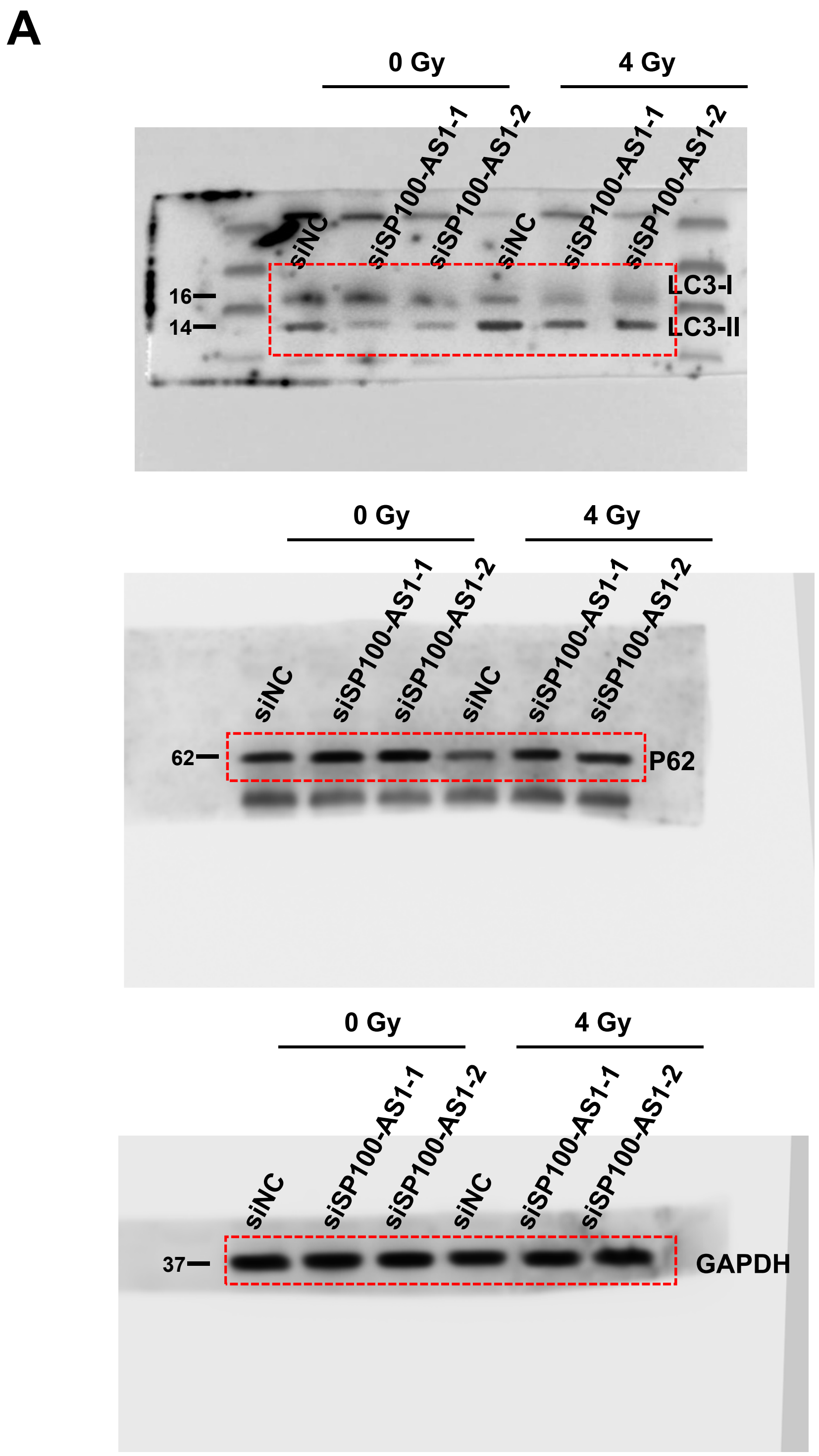
A
0 Gy
4 Gy
siNC
siSP100-AS1-1
siSP100-AS1-2
siNC
siSP100-AS1-1
siSP100-AS1-2
16
14
LC3-I
LC3-II
0 Gy
4 Gy
siNC
siSP100-AS1-1
siSP100-AS1-2
siNC
siSP100-AS1-1
siSP100-AS1-2
62
P62
0 Gy
4 Gy
siNC
siSP100-AS1-1
siSP100-AS1-2
siNC
siSP100-AS1-1
siSP100-AS1-2
37
GAPDH

Figure 5A

A

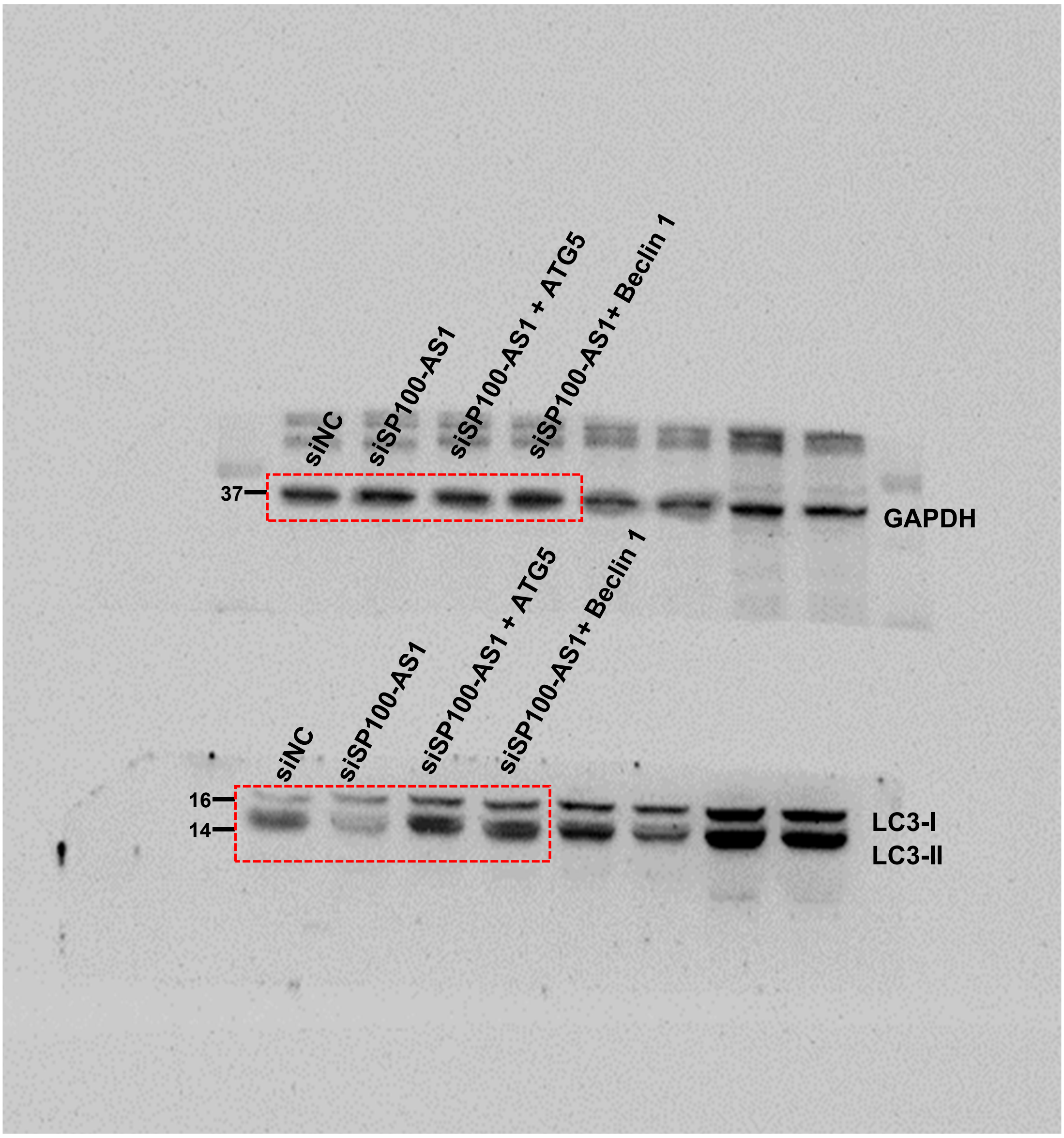

siNC
siSP100-AS1
siSP100-AS1 + ATG5
siSP100-AS1+ Beclin 1
37
GAPDH
siNC
siSP100-AS1
siSP100-AS1 + ATG5
siSP100-AS1+ Beclin 1
16
14
LC3-I
LC3-II

Figure 6E-6H

E

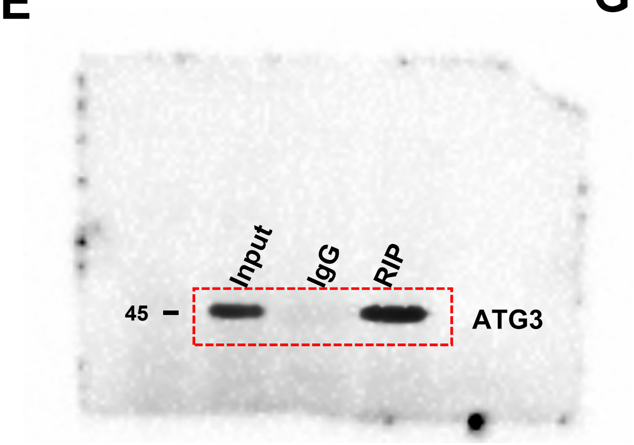

G

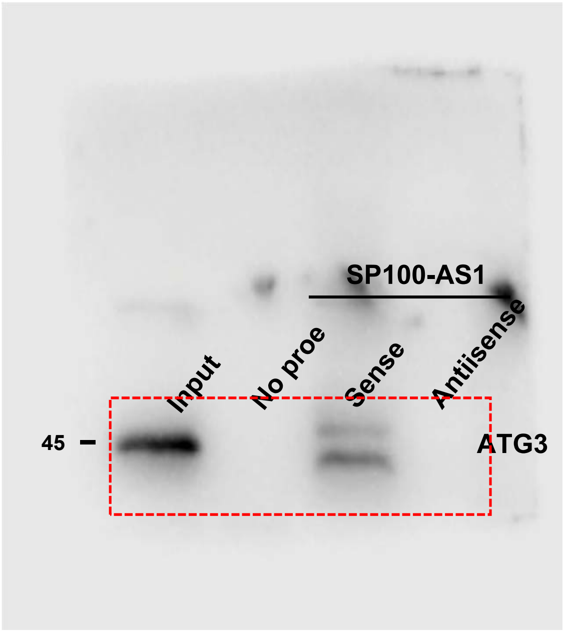

H

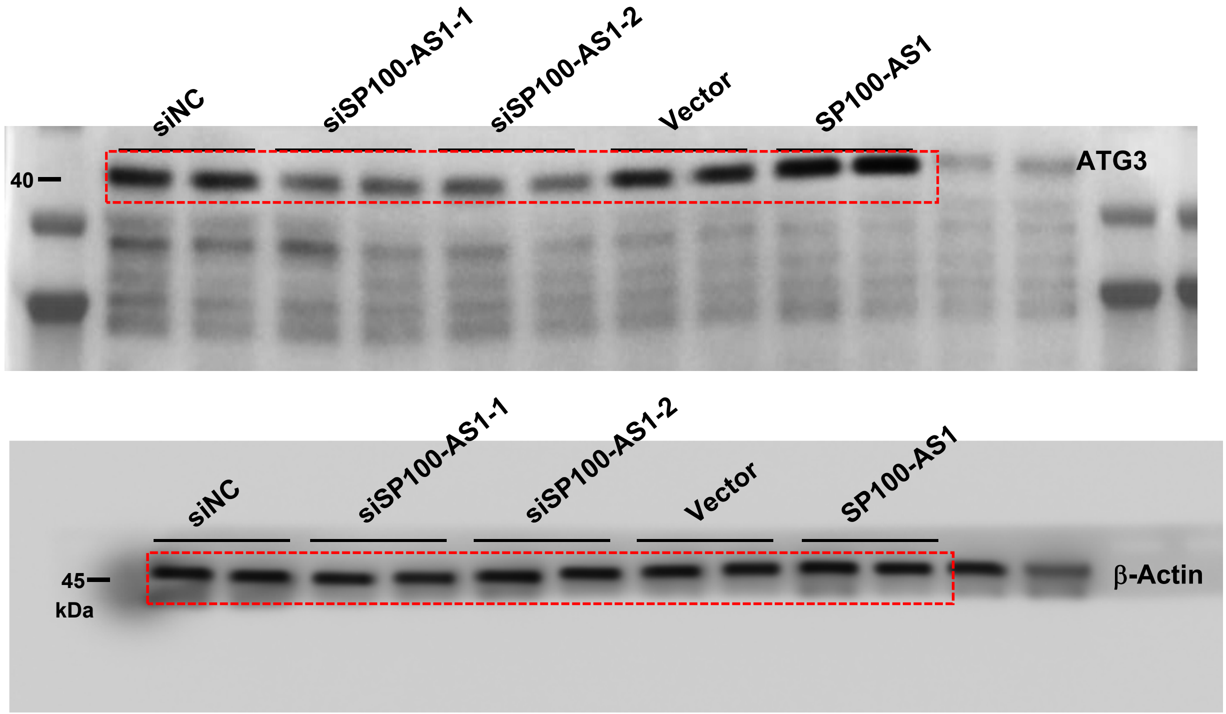

Figure 6I-6J

I

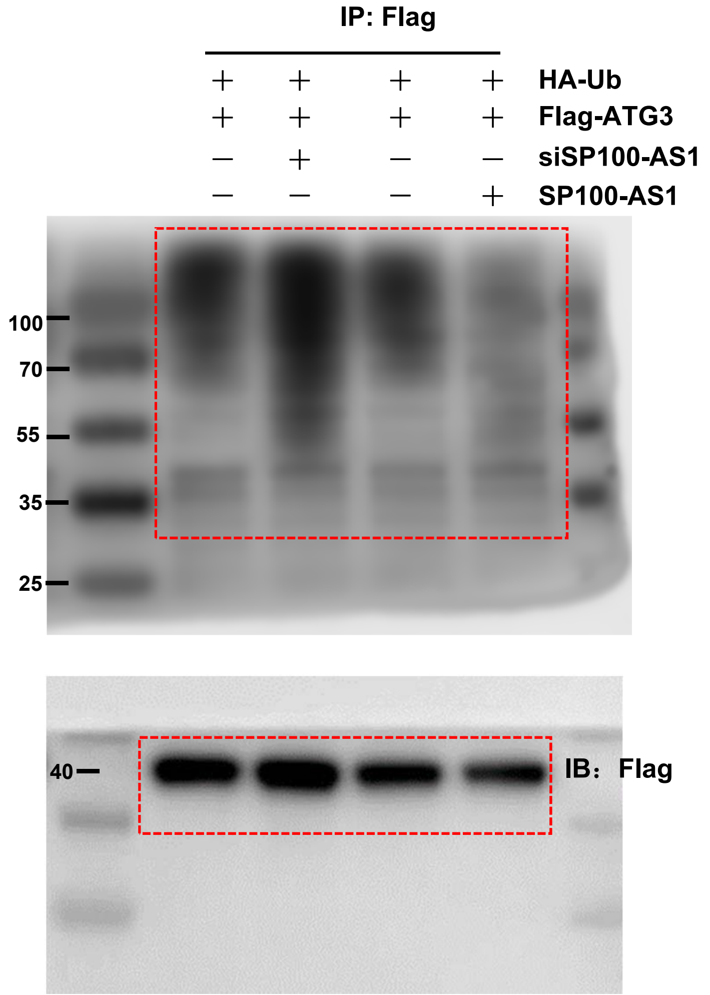

J

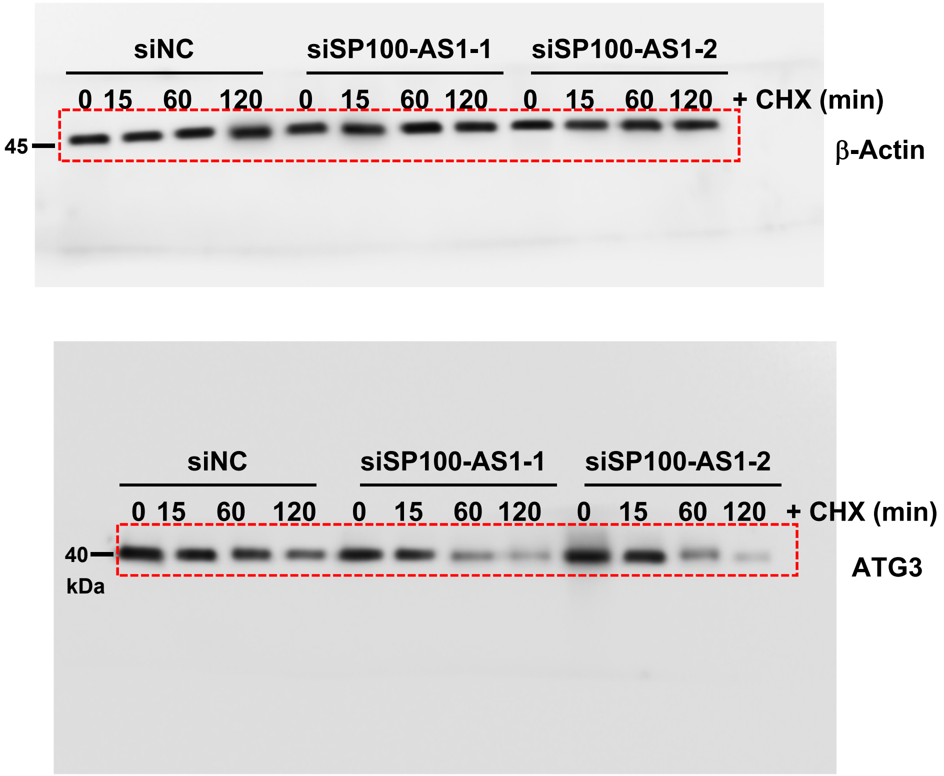

Figure 6K

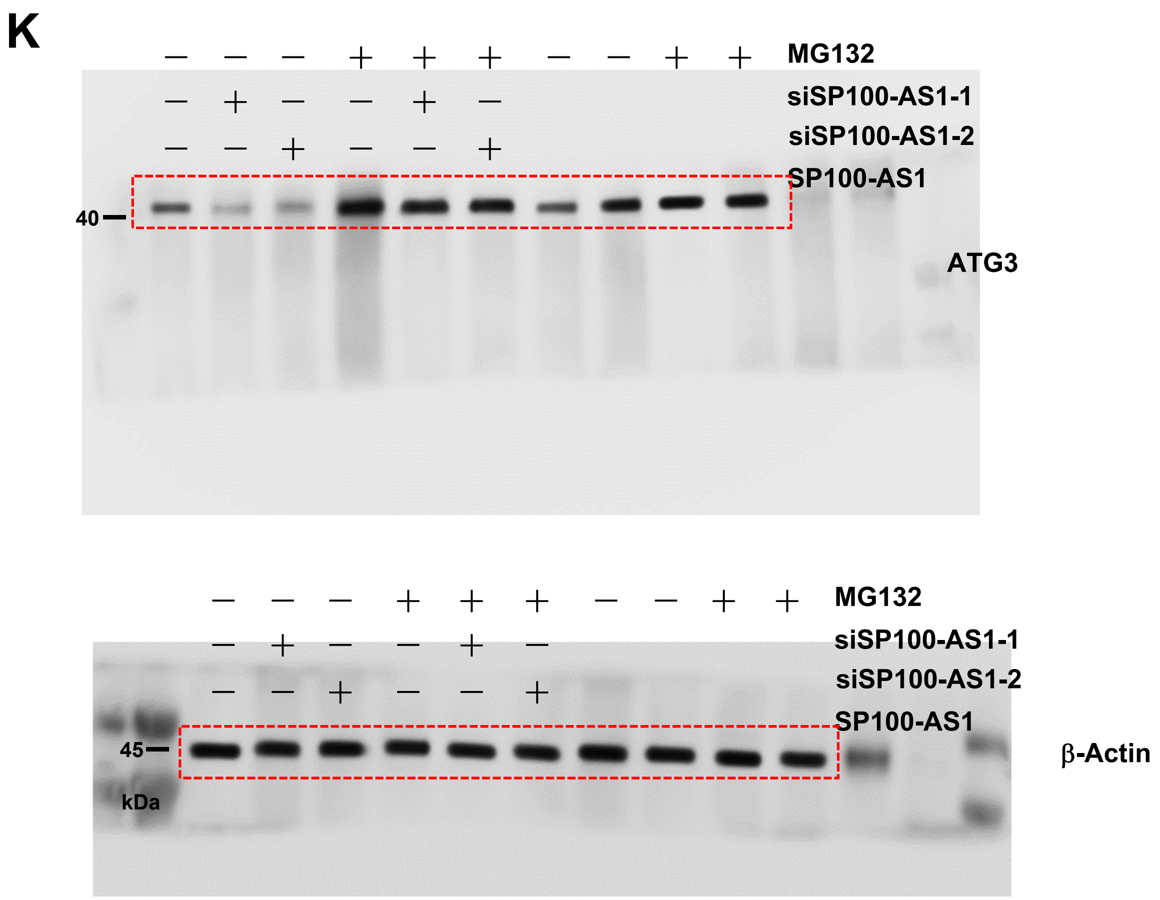
K
MG132
siSP100-AS1-1
siSP100-AS1-2
SP100-AS1
40
ATG3
MG132
siSP100-AS1-1
siSP100-AS1-2
SP100-AS1
45
kDa
β-Actin

Figure 8

A

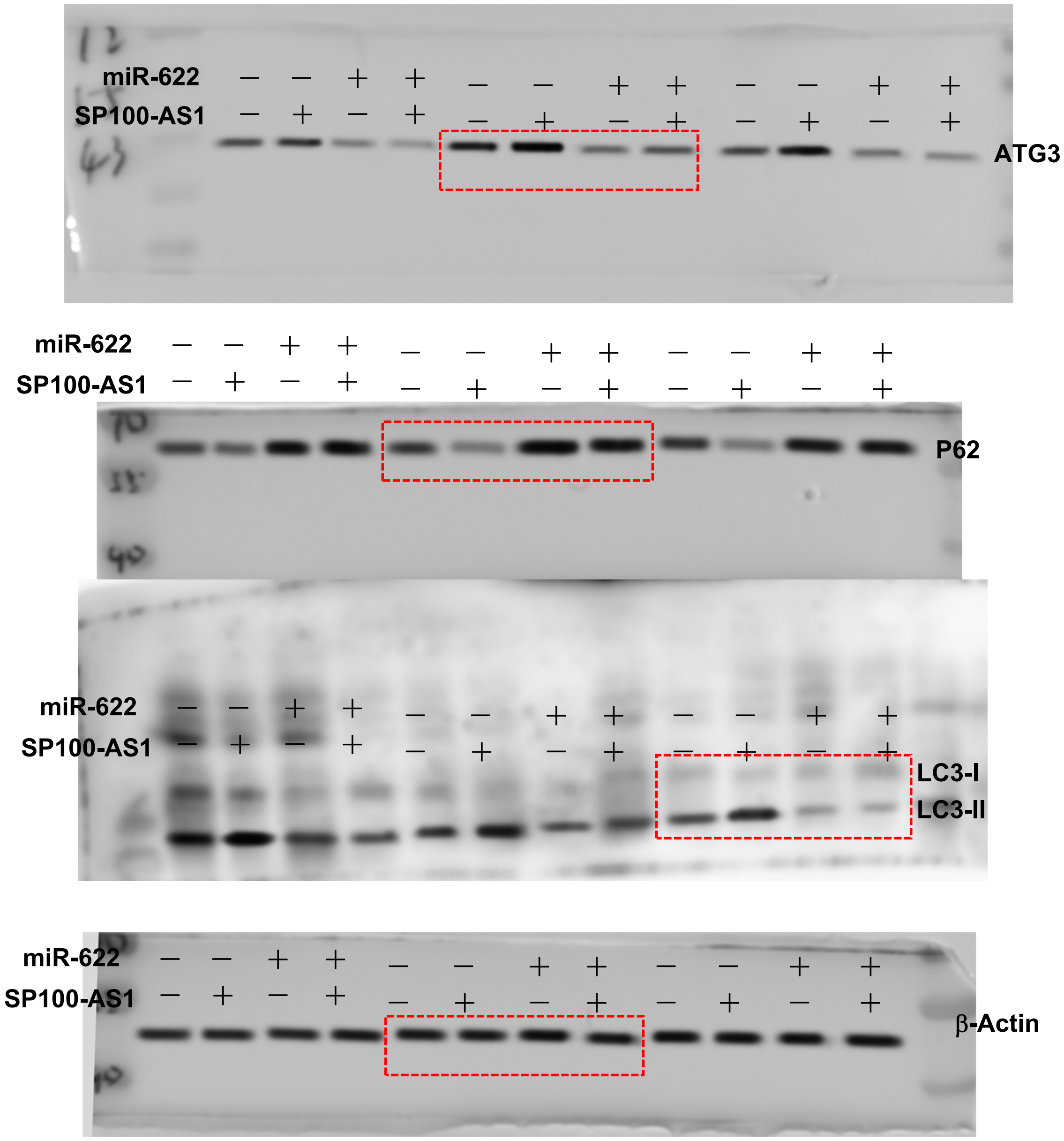
miR-622
SP100-AS1
ATG3
P62
LC3-I
LC3-II
β-Actin

E

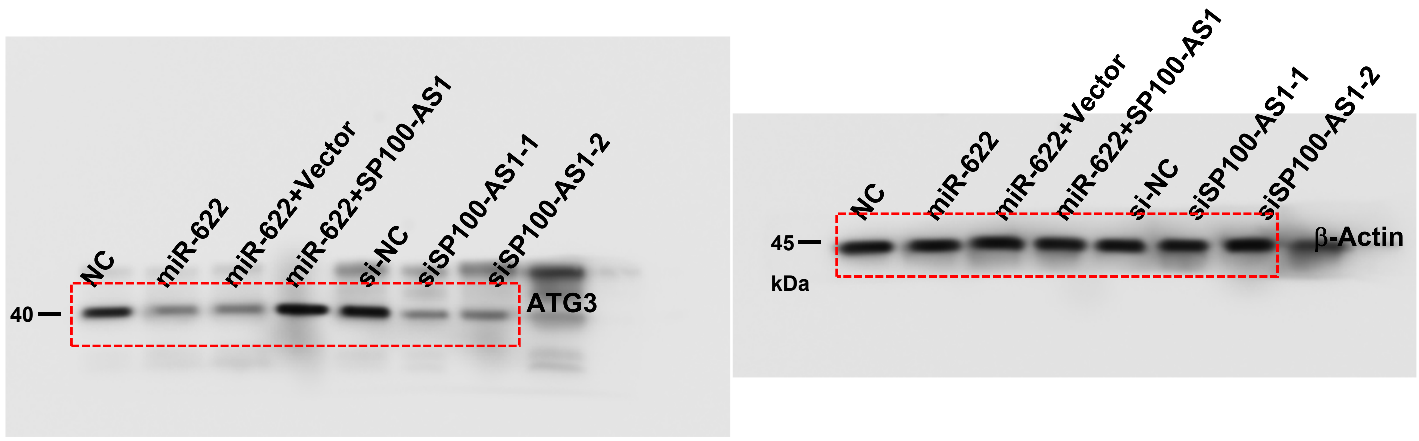
NC
miR-622
miR-622+Vector
miR-622+SP100-AS1
si-NC
siSP100-AS1-1
siSP100-AS1-2
40
ATG3
45
kDa
β-Actin

Figure S2

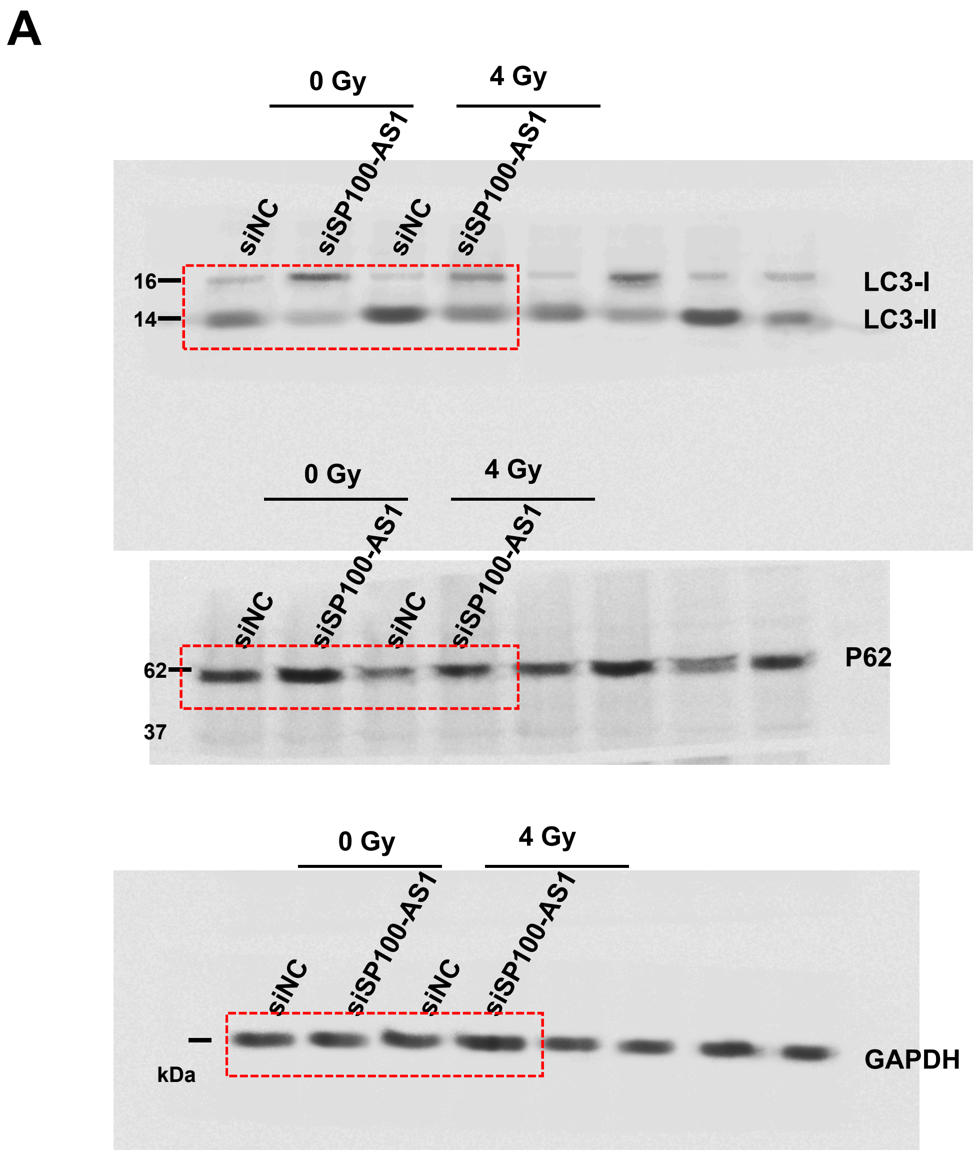
A
0 Gy
4 Gy
siNC
siSP100-AS1
siNC
siSP100-AS1
16
14
LC3-I
LC3-II
0 Gy
4 Gy
siNC
siSP100-AS1
siNC
siSP100-AS1
62
37
P62
0 Gy
4 Gy
siNC
siSP100-AS1
siNC
siSP100-AS1
kDa
GAPDH

Figure S3

A

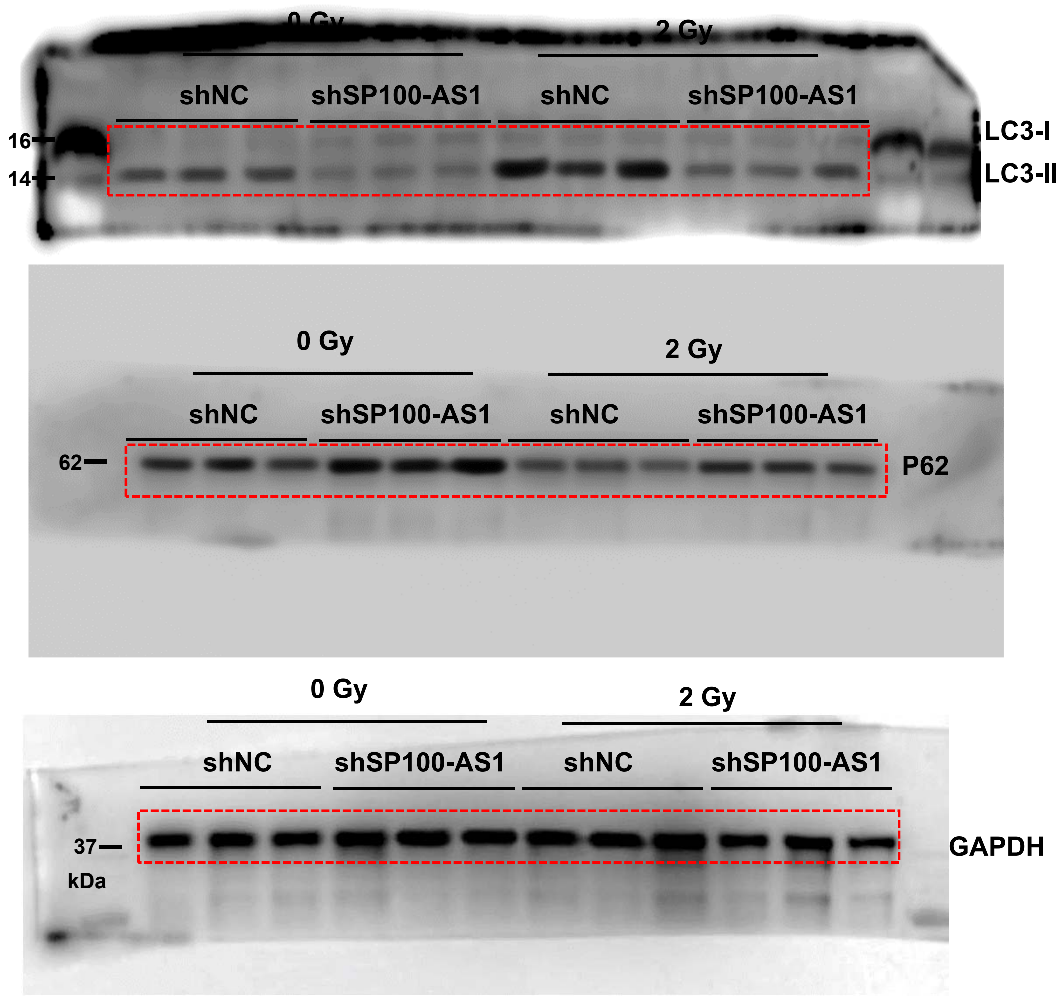
0 Gy
2 Gy
shNC
shSP100-AS1
shNC
shSP100-AS1
16
14
LC3-I
LC3-II
0 Gy
2 Gy
shNC
shSP100-AS1
shNC
shSP100-AS1
62
P62
0 Gy
2 Gy
shNC
shSP100-AS1
shNC
shSP100-AS1
37
kDa
GAPDH

Figure S4

A

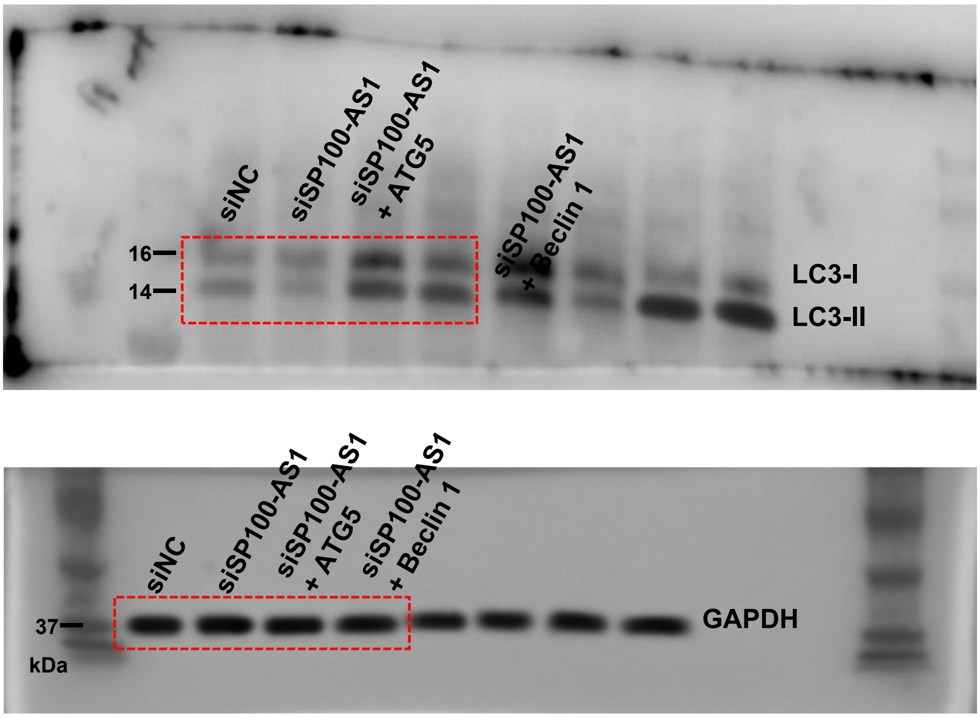
siNC
siSP100-AS1
siSP100-AS1
+ ATG5
siSP100-AS1
+ Beclin 1
16
14
LC3-I
LC3-II
siNC
siSP100-AS1
siSP100-AS1
+ ATG5
siSP100-AS1
+ Beclin 1
37
kDa
GAPDH
