# Supplementary material for: A novel long noncoding RNA SP100-AS1 induces radioresistance of colorectal cancer via sponging miR-622 and stabilizing ATG3
Source: Cell Death Differ. 2022 Aug 17;30(1):111–24. doi: 10.1038/s41418-022-01049-1 (PMC9883267; doi:10.1038/s41418-022-01049-1)
Supplement: Supplementary file 10 — CDD-21-2807RR-Author-Contribution-Form [file 41418_2022_1049_MOESM10_ESM.pdf]

DECLARATION OF CONTRIBUTIONS TO ARTICLE

ADMC

Manuscript Number:	Journal Name:	
CDD-21-2807RR	*Cell Death & Differentiation*	(the 'Journal')

Proposed Title of the Contribution:

A novel long noncoding RNA SP100-AS1 induces radioresistance of colorectal cancer via sponging miR-622 and stabilizing ATG3 (the 'Contribution)

Author(s):

You Zhou, Yingjie Shao, Wenwei Hu, Jinping Zhang, Yufang Shi, Xiangyin Kong, Jingting Jiang (the 'Authors')

For all *CDD* articles, each person named as an author in the published version must be able to show he or she has contributed substantially to the article.

Authorship credit should be based on 1) substantial contributions to conception and design, acquisition of data, or analysis and interpretation of data; 2) drafting the article or revising it critically for important intellectual content; and 3) final approval of the version to be published. Authors should meet conditions 1, 2 and 3.

Any person who cannot be shown to have made a substantial contribution to the article cannot be listed as an author in the final version. The name of any person who is deemed to have made a minor contribution can, however, appear in the Acknowledgments section of the article.

Please complete the table below to indicate the contributions of all named authors to the manuscript.

Author Full Name:	Specification of Contribution to the Manuscript:
You Zhou	Dr. You Zhou conceived of the study, participated in its design and performed all experiments.
Yingjie Shao	Dr. Yingjie Shao collected tissue samples and the clinical data.
Wenwei Hu	Dr. Wenwei Hu analyzed and interpreted the data.
Jinping Zhang	Dr. Jinping Zhang analyzed and interpreted the data.
Yufang Shi	Prof. Yufang Shi participated in study design and revised the manuscript.
Xiangyin Kong	Prof. Xiangyin Kong revised the manuscript.
Jingting Jiang	Prof. Jingting Jiang conceived of the study and participated in its design.

Please complete the table below to indicate the contributions of all named authors to the figures.

Figure 1:

You Zhou, Yingjie Shao, Jingting Jiang

Figure 2:

You Zhou,Yingjie Shao, Jingting Jiang

Figure 3:

You Zhou,Wenwei Hu, Jingting Jiang

Figure 4:

You Zhou,Wenwei Hu, Jingting Jiang

Figure 5:

You Zhou, Jingting Jiang

Figure 6:

You Zhou, Jingting Jiang

Signed for and on behalf of the Author(s):	Print Name:	Date:
	Jingting Jiang	28th July, 2022
